# The role of combination therapy in the treatment of severe infections caused by carbapenem resistant gram-negatives: a systematic review of clinical studies

**DOI:** 10.1186/s12879-021-06253-x

**Published:** 2021-06-09

**Authors:** Alessia Savoldi, Elena Carrara, Laura J. V. Piddock, Francois Franceschi, Sally Ellis, Margherita Chiamenti, Damiano Bragantini, Elda Righi, Evelina Tacconelli

**Affiliations:** 1 Division of Infectious Diseases, Department of Diagnostic and Public Health, University of Verona, P.Le L.A. Scuro 10, 37134 Verona, Italy; 2 Global Antibiotic Research & Development Partnership (GARDP), 15 Chemin Louis-Dunant, Geneva, Switzerland; 3 Division of Infectious Diseases, Department of Internal Medicine I, German Center for Infection Research, University of Tübingen, Otfried Müller Straße 12, 72074 Tübingen, Germany; 4 German Centre for Infection Research (DZIF), Clinical Research Unit for Healthcare Associated Infections, Tübingen, Germany

**Keywords:** Carbapenem resistant gram negative, Systematic review, Combination of antibiotics, Antibiotic treatment

## Abstract

**Background:**

Effective treatment of sepsis due to carbapenem-resistant Gram-negative bacteria (CR-GNB) remains a challenge for clinicians worldwide. In recent years, the combination of antibiotics has become the preferred treatment strategy for CR-GNB infection. However, robust evidence to support this approach is lacking. This systematic review aimed at critically evaluating all available antibiotic options for CR-GNB sepsis with particular focus on combination.

**Methods:**

We systematically searched published literature from January 1945 until December 2018 for observational comparative and non-comparative studies and randomized trials examining any antibiotic option for CR-GNB. Studies were included if reporting microbiologically-confirmed infection caused by Acinetobacter baumannii, Enterobacteriaceae/Klebsiella spp., or *Pseudomonas aeruginosa*, reporting at least one of the study outcomes, and definitive antibiotic treatment. Carbapenem-resistance was defined as phenotypically-detected in vitro resistance to at least one of the following carbapenems: doripenem, ertapenem, imipenem, meropenem. Each antibiotic regimen was classified as “defined” when at least the molecular class(es) composing the regimen was detailed. Primary outcomes were 30-day and attributable mortality. Bayesian network meta-analysis (NMA) approach was selected for quantitative synthesis to explore feasibility of pooling data on antibiotic regimens.

**Results:**

A total of 6306 records were retrieved and 134 studies including 11,546 patients were included: 54 studies were on Acinetobacter, 52 on Enterobacteriaceae/Klebsiella, 21 on mixed Gram-negative, and 7 on Pseudomonas. Nine (7%) were RCTs; 19 prospective cohorts (14%), 89 (66%) retrospective, and 17 (13%) case series. Forty-one studies (31%) were multicentric. Qualitative synthesis showed an heterogeneous and scattered reporting of key-clinical and microbiological variables across studies. Ninety-two distinct antibiotic regimens were identified with 47 of them (51%, 5863 patients) not reporting any details on numbers, type, dosage and in vitro activity of the included antibiotic molecules. The NMAs could not be performed for any of the selected outcome given the presence of too many disconnected components.

**Conclusion:**

The existing evidence is insufficient to allowing for the formulation of any evidence-based therapeutic recommendation for CR-GNB sepsis. Future studies must provide a standardized definition of antibiotic regimen to drive recommendations for using combination of antibiotics that can be reliably applied to clinical practice.

**Supplementary Information:**

The online version contains supplementary material available at 10.1186/s12879-021-06253-x.

## Introduction

Carbapenem-resistant Gram-negative bacteria (CR-GNB) can cause a range of serious infections in hospitalised patients, requiring the prompt initiation of antibiotic treatment [1]. Currently, the availability of active antibiotic options is very limited because of the relentless increase of extensively drug-resistant (XDR) and pan drug-resistant (PDR) strains [2, 3]. In recent years, only a few new agents have been granted approval for the treatment of CR-GNB infections; however, evidence on their efficacy against specific bacterial phenotypes is still scarce. Additionally, none of these new agents belongs to new antibiotic classes and the development of resistance has been reported after clinical use [4, 5].

The severity of those clinical scenarios, combined with the constant uncertainty around antibiotics’ efficacy in terms of clinical and microbiological outcomes, has led clinicians to use combinations of multiple agents as a preferred strategy for treating CR-GNB infections in clinical practice. There are only a handful of guidelines supporting this choice and they are based on low-quality evidence [6, 7]. Moreover, due to the inadequacy of available studies, recommendations are usually generic without clearly specifying type and number of agents that should be used in combination [8, 9]. As a consequence, clinicians are prescribing a wide range of treatment regimens in their current practice with no proven efficacy and potential side effects from both patient- and stewardship-perspectives.

This study was conducted as part of the COHERENCE project, a Global Antibiotic Research & Development Partnership (GARDP) initiative aimed at summarizing evidence and common practice relative to the use of combination therapy for treating severe infections caused by CR-GNB. We report the results of the systematic review of the literature on available antibiotic options for the treatment of infections caused by CR-GNB (Acinetobacter, Pseudomonas and Enterobacteriaceae).

## Methods

### Study selection and eligibility criteria

Studies on adult and paediatric populations with sepsis caused by CR-GNB (Acinetobacter baumannii., Enterobacteriaceae, *Pseudomonas aeruginosa*) receiving any type of antibiotic treatment after results of in vitro susceptibility testing were included. Comparative and non-comparative observational studies, randomized control trials (RCTs) and case series were considered eligible. Inclusion criteria were: studies reporting microbiologically-confirmed infections; studies reporting outcomes on CR-GNB without any further classification or on at least one of the following CR bacteria: A. baumannii, Enterobacteriaceae/Klebsiella spp., *P. aeruginosa*; studies reporting data on targeted antibiotic treatment. Exclusion criteria were: studies reporting data exclusively on empiric treatment; studies reporting data on suspected infections without microbiological confirmation; studies not reporting carbapenem-resistance confirmed by phenotypic result; studies focusing only on colonization; studies reporting data on non-bacteraemic urinary tract infection; case reports, and editorials.

#### Definitions and outcomes

Carbapenem-resistance was defined as phenotypically-detected in vitro resistance of the isolate to at least one of the following carbapenems: doripenem, ertapenem, imipenem, meropenem in accordance with the diagnostic assay and the breakpoint reference system adopted by the individual study. The primary outcomes were 30-day all-cause mortality and attributable mortality. If 30-day mortality was not available, all-cause mortality at any time-point was extracted. Secondary outcomes were: clinical cure, microbiological cure, length of hospital stay (LOS), re-hospitalization, resistance development after antibiotic treatment and any adverse events. Attributable mortality was defined as death due to CR-GNB infection as reported by the study. Re-hospitalization was defined as new hospital admission due to infection within 30–90 days from the initial episode. With regard to the definitions of clinical cure, microbiological cure, LOS, and costs, the definition provided by each study was accepted. Underlying comorbidities were defined as follows: pulmonary disease, including chronic pneumopathy and/or chronic obstructive pulmonary disease; neurological disease, including any acute or chronic neurological condition; renal disease, including any degree of acute/chronic renal impairment, and/or ongoing dialysis. The definition of immunocompromised state was fulfilled by the presence of one of the following conditions: long-term steroid therapy, haematological malignancy, neutropenic state. Each antibiotic regimen was classified as “defined antibiotic regimen” when at least the antibiotic classes (i.e. carbapenem plus polymyxin) composing the regimen was clearly specified. Since the definition of sepsis has been changed several times over the included study period, the definition of sepsis provided by each individual study was accepted.

#### Search strategy

A comprehensive search of peer-reviewed literature was carried out by searching the following electronic databases: PubMed, Cochrane Library, and Clinicaltrials.gov starting from January 1945 until December 2018. PubMed was searched by conducting three separate search strategies according to selected bacterial phenotype as follows: A. baumannii, Enterobacteriaceae/Klebsiella spp., *P. aeruginosa*. The search was not restricted by language, year of publication or study design. Additionally, references to the included studies were systematically screened for the inclusion of relevant articles. The search strings are reported in Table S1.

#### Data extraction

Two reviewers (A.S. and E.C.) screened independently the title/abstract of all publications. Subsequently, the same reviewers examined the full-texts of all the potentially eligible records passing the first search step for eligibility. Inconsistencies and discrepancies were discussed and resolved after the achievement of agreement between the two reviewers or by involving a third reviewer (E.T). The following variable domains were entered into a pre-defined excel database: study-related variables (authors, year of publication, study country and design, setting), patient-related variables (age, sex, underlying comorbidities), infection-related variables (severity score, sepsis/septic shock, infection source) and treatment-related variables (number and type of agents, route of administration, dosage, duration).

#### Quality appraisal

Risk of bias (RoB) for individual studies was assessed by two independent reviewers (A.S. and E.C.) using Cochrane RoB tool for RCTs [10] and an adapted version of RoB (Risk Of Bias tool for Non-randomized Studies of Interventions ROBINS-I) [11] for observational studies. Discrepancies between the two reviewers were clarified by discussion or by involving a third reviewer (E.T.). For both RoB tools, each domain was assigned low, moderate, serious, unknown (if no sufficient information). Detailed description of the tools employed for assessment of the study quality is displayed in Table S2.

#### Data synthesis and analysis

The results of the systematic review were stratified according to the following bacterial phenotypes: A. baumannii, Enterobacteriaceae, *P. aeruginosa*, and Mixed Gram-negative, in case of the individual study reported data on aggregated CR-GNB without any further classification by species. Continuous and categorical variables were reported as median and median frequency with interquartile range (IQR), respectively. Given that considerable heterogeneity among treatment schemes was expected, with very few studies performing a direct pairwise comparison, we planned to explore the feasibility of pooling comparative data on the various antibiotic treatment schemes using Bayesian network meta-analysis (NMA) (STATA command “network setup”). We planned to assess heterogeneity in each pairwise comparison with I^2^ test, and the inconsistency between direct and indirect evidence using Wald Test (within the whole network) and node-splitting (within each specific comparison). For the quantitative analysis, we included exclusively patients receiving defined antibiotic regimens. Treatment observation was adopted as the unit of analysis. All statistical analyses were carried out with STATA version 15 (Statacorp LLC, Texas, United States). The methods were based on the framework provided by the Preferred Reporting Items for 2015 Systematic Review and Meta-Analysis Protocols for NMA (PRISMA-NMA) checklist [12]. This study was registered on the International Prospective Register of Systematic Reviews (PROSPERO) with the following trial number: CRD42019127928 dated 9 April 2019 (https://www.crd.york.ac.uk/prospero/display_record.php?RecordID=127928).

## Results

### Study selection and characteristics

A total of 6306 records were retrieved from the search. After applying inclusion and exclusion criteria, 134 studies were included in the review. The flowcharts of the study selection process are in Figures S1a, b, c. The list of the included studies is provided in Annex V. Overall, the 134 studies were conducted in 19 countries and involved 11,546 patients (Fig. S2). Stratifying by bacterial phenotype, 54 (40%) studies (4395, 38% patients) reported data on A. baumannii, 52 (38.5%) studies (4118, 35.5% patients) reported data on Enterobacteriaceae, while only seven (5%) studies (545, 5% patients) specifically reported data on *P. aeruginosa*. Twenty-one (16.5%) studies (2488, 21.5% patients) reported data on unspecified mixed Gram-negative. Eighty-nine (66%) studies had a retrospective design, whereas 19 (14%) and 17 (13%) were case series and prospective, respectively. Nine (7%) were randomized studies. Ninety-three (69%) studies were conducted in a single hospital, while the remaining 41 (31%) studies were multicentric. With regard to the study setting, 81 (61%) studies were performed across hospital wards, while the remaining 53 (39%) were conducted in an Intensive Care Unit (ICU) setting.

### Patient and infection characteristics

#### Adult patients

One hundred and thirty-one (98%) studies focused on adults, including a total of 11,472 hospitalized patients (4391 Acinetobacter, 4109 Enterobacteriaceae, 545 Pseudomonas, 2488 Mixed gram-negative). The median patient age was 59.5 years (IQR 51–65) and the median Apache II score was 20 (IQR 17–23). Only 18 (14%) studies reported all the listed underlying comorbidities. The median frequency of immunocompromised status was 26% (IQR 14–40). Sixty-seven (51%) studies clearly defined the source of the infection. Among those, bloodstream infections were the most common (37/131 studies, 28%), followed by hospital- or ventilator acquired pneumonia (HAP/VAP) (27/131 studies, 20%). Information on monomicrobial/polymicrobial infection was reported in 79 (60%) studies. Overall, 35 studies (26%) had monomicrobial infections as a specific inclusion criterion. Data on appropriateness of empiric antibiotic treatment, based on in vitro susceptibility pattern, was available in 30/131 studies (23%) with a median value of 48% (IQR 22–67). Patient and infection characteristics stratified by bacterial phenotype are shown in Table 1.

**Table 1:** Demographic and clinical characteristics of adult patients

Patient and infection characteristics	Bacterial phenotype				Total 131 studies
	A. baumannii 53 studies	***P. aeruginosa*** 7 studies	Enterobacteriaceae 51 studies	Mixed Gram- negative 20 studies	
**Age years,** median (IQR) (103 studies provided data)	57 (51–65)	59.5 (46–63)	60 (51–64)	59.5 (51–65)	59.5 (51–65)
**Pulmonary illness**	20 (10–26)	19 (15–28)	16 (10–23)	21 (12–25)	20 (11–26)
Median frequency % (IQR) (64 studies provided data)					
**Neurological illness**	13 (8–22)	–	22 (9–24)	20 (11–28)	17 (9–26)
Median frequency % (IQR) (36 studies provided data)					
**Renal illness**	23 (18–35)	23 (20–35)	24.5 (22–36)	20 (18–33)	22 (18–35)
Median frequency % (IQR) (76 studies provided data)					
**Immunocompromised status**	26 (14–44)	31 (15–41)	29.5 (18–47)	32 (15–43)	26 (14–40)
Median frequency % (IQR) (72 studies provided data)					
**Infection type,** n (%)					
Mixed infection	18 (35)	4 (57)	31 (61)	11 (57)	64 (49)
Bloodstream infection	13 (24)	0	18 (35)	6 (28.5)	37 (28)
Ventilator−/Hospital acquired pneumonia	20 (37)	3 (43)	2 (4)	2 (9.5)	27 (21)
Other^a^	2 (4)	0	0	1 (5)	3 (2)
**Apache II score**	18 (15–22)	14 (14–16)	20 (15–21)	19 (19–23)	20 (17–23)
Median (IQR) (76 studies provided data)					
**Monomicrobial, n** (%) (79 studies provided data)	13 (26)	3 (43)	12 (23)	6 (27)	34 (26)
**Appropriate empiric treatment°**	–	–	–	–	48 (22–67)
Median frequency % (IQR)^b^ (30 studies provided data)					

^a^Other: Central nervous system infections in A. baumannii group; mediastinitis in Mixed Gram-negative group

^b^Given that the paucity of studies providing data, only overall value was computed

*IQR* Interquartile range

#### Paediatric patients

Three studies (2%) reported data on 74 paediatric patients (four on Acinetobacter, nine on Enterobacteriaceae, 61 on Mixed Gram-negative). None of these studies reported any clinical characteristics. A summary of the studies is provided in Table S3.

### Microbiological characteristics

One hundred and seven studies (80%) specifically referred to standardized breakpoint reference systems for defining carbapenem resistance; among them, 84 studies (63%) referred to The Clinical & Laboratory Standards Institute and 23 studies (17%) referred to the European Committee on Antimicrobial Susceptibility Testing. One hundred one (76%) studies specified the microbiologic tool used for detecting carbapenem resistance. Among the six different methods employed, automated microdilution was the most common (48 studies, 36%), followed by E-test (22 studies, 16%) and disc diffusion (17 studies, 13%), (Table S4). The molecular mechanism causing resistance to carbapenems was specified in 39 studies (28%), especially in the Enterobacteriaceae group, for which the most frequent mechanism of resistance was the production of KPC type carbapenemase (Table S5). Quantitative data on concomitant prevalence of resistance to other antibiotic agents in CR-GNB was reported in 79/134 (58%) studies. The highest resistance levels (above 50%) for other antibiotic classes were frequently detected for aminoglycosides and polymyxins classes. The distribution (percentages) of the concomitant resistance by study is displayed in Figure S3.

### Antibiotic treatment regimens

**Fig. 1:**
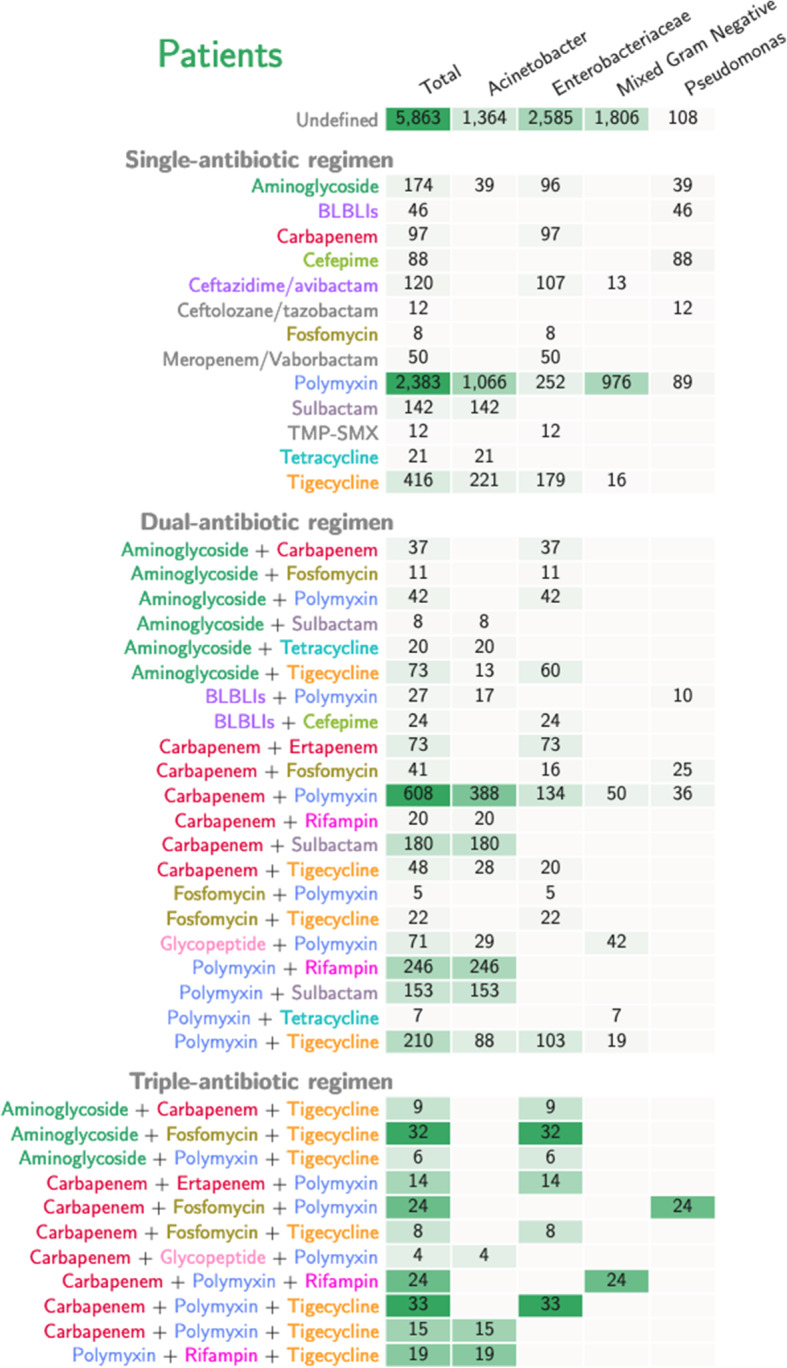
Antibiotic regimens assessed in the included studies stratified by bacterial phenotype and number of patients. Legend: The antibiotics belonging to same classes are grouped. Carbapenem classes includes Group A carbapenem (doripenem, imipenem, meropenem). Tigecycline is the only agent belongs to the class of glycyclyine. Sulbactam was grouped separately for Acinetobacter. The computation of patients referred to the outcome mortality (or clinical cure, if mortality was not reported by the individual study). In case of multiple outcomes, the number of patients for each antibiotic regimen was computed for only one outcome. BLBLIs: beta lactam-beta lactam inhibitors

Overall, 92 distinct antibiotic regimens were used. Forty-five out of 92 (49%) antibiotic regimens accounting for use in 5683 (49.4%) patients were classified as “defined antibiotic regimen”. Among them, 13 were single-, 21 were dual, and 11 were triple-antibiotic regimens. Polymyxin was the most prescribed antibiotic class both alone and in combination for each bacterial phenotype. Single antibiotic regimen with a polymyxin was used in 2383 patients. Nine out of 11 dual-antibiotic regimens included a polymyxin. The most frequently prescribed dual antibiotic regimens were: polymyxin plus carbapenem (608 patients), followed by carbapenem plus rifampin (246 patients) and polymyxin plus tigecycline (210 patients). The distribution of the antibiotic regimens grouped by patients and bacterial phenotype is detailed in Fig. 1.

The remaining 5863 (50.6%) patients were treated with 47 (51%) different “undefined antibiotic regimens”. In this group, the definition of “antibiotic combination” was very generic and extremely heterogeneous, including a variable number and type of antibiotic agents (i.e. carbapenem-based, colistin-sparing, tigecycline-containing, any combination). For 42/47 of these antibiotic combinations, the in vitro susceptibility of the included agents was unknown.

### Outcomes description

The 134 studies measured seven different outcomes. The most tested outcome was mortality (118 studies, 87%): 30-day mortality was reported in 65 (48%) studies, while attributable mortality was reported in 19 (14%) studies. Clinical cure and microbiological cure were also frequently tested by the individual studies (66 and 62 studies, 48 and 46%). Full results stratified by bacterial phenotype are displayed in Table 2.

**Table 2:** Outcomes assessed by the included studies clustered by bacterial phenotype

Outcome	Bacterial phenotype				Total 134 studies
	A. baumannii 54 studies	P. aeruginosa 7 studies	Enterobacteriaceae 52 studies	Mixed Gram-negative 21 studies	
**Mortality**					
30-day mortality	29	3	24	9	65
Mortality at other time point^a^	10	2	16	7	35
Attributable mortality	11	1	3	4	19
**Adverse effects**					
Renal	21	2	9	13	45
Diarrhea/C. difficile infection	–	–	4	3	7
Neurological	8	1	2	6	17
Not specified	3	3	3	3	12
Clinical cure	38	6	19	13	76
Length of hospital stay	4	–	2	1	7
Microbiological cure	37	4	13	11	65
Resistance development	7	–	3	1	11
Re-hospitalization	–	–	–	1	1

^a^Mortality at any time point other than 30-day and in-hospital mortality

### Quantitative synthesis

**Fig. 2:**
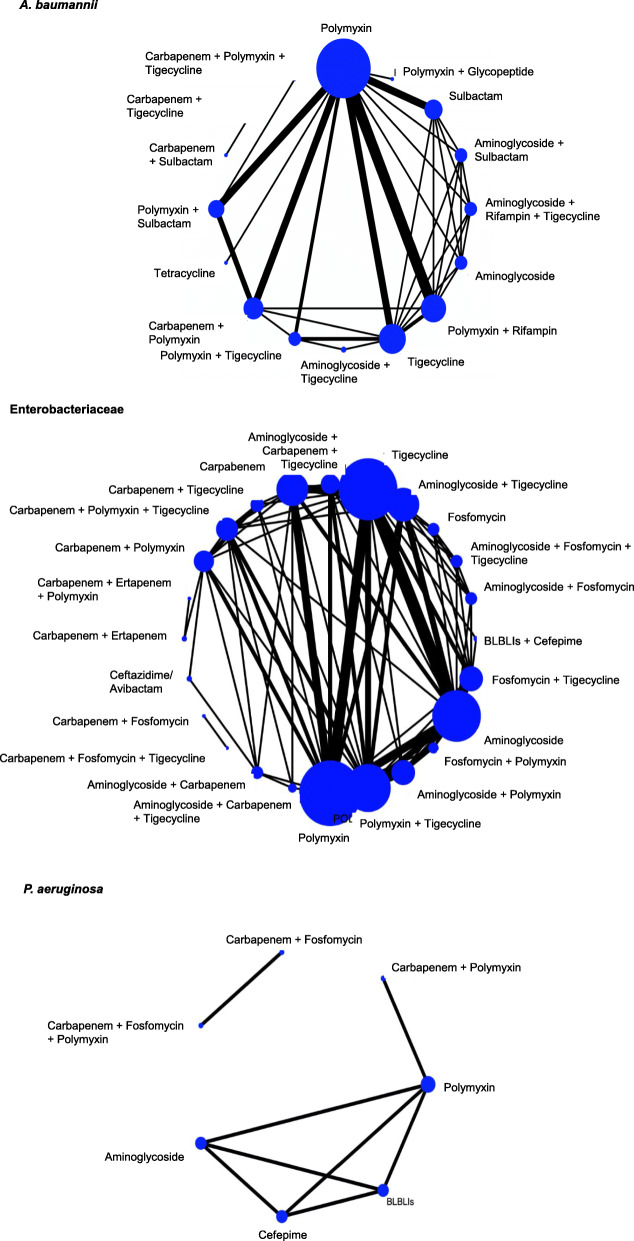
Network geometry of outcome mortality assessed for each bacterial phenotype

Only the 5683 patients receiving a “defined antibiotic regimen” were included in the quantitative synthesis. Four separate NMAs were run for the outcomes mortality, clinical cure, microbiological cure and adverse events for A. baumannii, Enterobacteriaceae and *P. aeruginosa*. Irrespective of the bacterial phenotype and the outcome considered, a quantitative analysis through NMA could not be performed because the network had too many disconnected components (i.e. the included regimens could be grouped in several independent components with no connections from one to the other). An example of the network geometry for the outcome mortality is displayed in Fig. 2. Outcome data from individual studies that were entered in the NMAs are reported in Annex III. Quantitative analyses for the remaining outcomes (LOS, re-admission, costs, resistance development and relapse of infection) were not conducted because of the scarcity of available observations (less than five for each bacterial phenotype).

### Quality appraisal

With regard to non-randomized studies, 119/125 (95%) of them had critical RoB concerning confounding, while 110/125 (88%) and 107/125 (86%) studies had critical RoB concerning selection of patients and classification of intervention, respectively. The majority of the domains pertaining to randomized studies were at moderate or low RoB. Table 3 summarises the quality assessment by domain. Full quality appraisal is shown in Table S6.

**Table 3:** Risk of Bias assessment of the studies by domain

Study type	NON-RANDOMIZED (125 studies)	Risk of bias, n (%)		
		CRITICAL	MODERATE	LOW
**Domain**	Confounding bias	119 (95)	6 (0.5)	0
	Bias in selection of participants	110 (88)	15 (1.2)	0
	Bias in classification of interventions	107 (86)	18 (14)	0
	Deviation from intended interventions	92 (74)	32 (26)	1 (0.8)
	Missing data	0	15 (12)	110 (88)
	Bias in selection of reported results	0	10 (8)	115 (92)
**Study type**	**RANDOMIZED (9 studies)**	**CRITICAL**	**MODERATE**	**LOW**
**Domain**	Selection bias	2 (22)	5 (55)	2 (22)
	Performance bias	2 (22)	6 (67)	1 (11)
	Detection bias	3 (33)	5 (55)	1 (11)
	Attrition bias	1 (11)	6 (67)	2 (22)
	Reporting bias	0	2 (22)	7 (77)
	Other bias	0	1 (1)	8 (6)

## Discussion

This systematic review provides a comprehensive and critical evaluation of the current evidence on the available antibiotic options for treating patients with sepsis due to CR A. baumannii, Enterobacteriaceae, and *P. aeruginosa*. Our results clearly revealed the challenges to developing evidence-based recommendations on the therapeutic management of severe infections caused by CR-GNB from the existing literature. The included studies showed a suboptimal reporting of key-variables, making results difficult to compare and ill-suited for adjusted analysis. Despite the availability of numerical data on 11,546 patients, no quantitative analyses could be performed because of the vast heterogeneity and the lack of clear definitions of the included antibiotic regimens.

The first part of our systematic review focused on a descriptive analysis of clinical and microbiological data. In patients with CR-GNB infections, especially those with critical illness, comorbidities and baseline severity of disease are known to be major contributors to the final outcomes [13]. However, our systematic review showed that only 21 comparative observational studies included an adjusted analysis for these confounders, whereas the remaining studies were generally too small to allow for adjustment. Overall, the studies had a median sample of 49, a figure which is considerably lower than the estimated sample of several hundreds or even thousands of patients that are needed for reliably assessing independent association of one antibiotic regimen with mortality in observational studies [6, 14].

The extremely wide range of outcomes selected by individual studies (i.e. four different definition of mortality were applied) could represent a further limitation and an additional source of heterogeneity when comparing and pooling data.

In terms of microbiological characteristics, causative pathogens varied in terms of species, mechanisms of resistance, minimum inhibitory concentrations thresholds to carbapenems, and pattern of concomitant resistance. This heterogeneity was common not only among different studies, but often within the same study. Overall, resistance to carbapenem was detected using five microbiological methods with various performance and accuracy [15].

Evidence from observational studies shows a survival benefit in patients with severe infections receiving in vitro active empirical treatment [16, 17]. However, a post hoc analysis of the only available RCT on the topic documented that early initiation of colistin did not impact on survival of patients with severe infections sustained by colistin-susceptible CR-GNB [18]. Similarly to other clinically relevant variables, data on empirical coverage from our systematic review were scarce (only 23% of the studies) and did not allow to conduct any further analysis.

Our systematic review included mostly low-quality observational studies. The included randomized controlled trials were generally of better quality, but did not contribute significantly to the overall analysis. In particular, the trials on new antibiotics showed important limitations related to the inclusion of very small sample size and the use of heterogeneous comparison groups [19, 20].

In the last years, several systematic reviews and meta-analyses have addressed the issue of the superiority of combination treatment, mainly focusing on polymyxin-carbapenem combination. Conclusions from these systematic reviews were generally undermined by methodological limitations of the included studies [6, 21, 22] and they did not match the results from a more recent RCT addressing the same clinical question [23]. Our systematic review included overall 97 distinct antibiotic regimens; this result reflects the lack of standardization in clinicians’ prescribing, as pointed out in the third paper of this series (reference to be added). Among these 92 regimens, less than a half were accurately defined. Most studies, in fact, adopted a generic definition of combination therapy frequently with unknown in vitro susceptibility, rather than defining specific combinations. In this way, patients could be easily allocated to the various treatment arms and potentially contribute to the retrospective assessment of determinants of mortality. However, in the wake of existing evidence and experts’ recommendations, we deemed those data as scarcely informative for clinical practice and we restricted the analysis to clearly defined antibiotic regimens [21].

The purpose of our quantitative analysis was to evaluate the effectiveness of defined antibiotic regimens in patients with sepsis due to CR A. baumannii, Enterobacteriaceae and *P. aeruginosa* in terms of clinical and microbiological outcomes. Given that several studies did not offer a direct comparison, we selected the NMA approach that can integrate both direct evidence (from studies directly comparing two antibiotic regimens) with indirect evidence (information on two antibiotic regimens derived via a common comparator), increase the precision in the estimates and produce a relative ranking of all antibiotic regimens for selected outcomes [24]. Unfortunately, the distribution of the patients across 45 defined antibiotic regimens did not allow the creation of a ‘connected network’ where all regimens are comparable via direct or indirect comparison. We could have forced the analysis or conducted separate NMAs for each network component, but potential results would have been again of poor clinical significance.

## Conclusion

In conclusion, in our systematic review we comprehensively collated evidence on all antibiotic options for treating sepsis due to CR-GNB. Besides providing a state of art of the quantity and the quality of current literature, our study has focused on the drawbacks in the definition of “combination therapy” and the implications that it might have in clinical practice. Methodological issues and inherent biases of existing studies have precluded a quantitative synthesis of results, not allowing identification of which is the optimal therapeutic strategy, as monotherapy or combination, for treating CR A. baumannii, Enterobacteriaceae or *P. aeruginosa* sepsis in specific clinical scenarios.

Although RCTs in this field are urgently needed to overcome many of the observed biases, they are challenging to conduct due to both operational and clinical difficulties, as recently proven by the CARE study, for example [19]. In this context, observational studies still play an important role if they fulfil specific requirements, such as prospective design, proper sample size, identification and adjustment for major confounders, outcome homogenization and accurate definition of the interventions [25, 26]. In particular, when referring to therapeutic interventions, the antibiotic regimens should be clearly specified in terms of in vitro activity, number, type, dosage and duration [25]. All these measures will provide high-quality evidence-based information that finally can guide the clinical practice in the near future.

## Supplementary Information

**Additional file 1: **ANNEX I: Methods. **Table S1.** Search strings adopted for the systematic review. **Table S2**: Description of the employed tools for assessment of the study quality. ANNEX II: Qualitative synthesis. **Figure S1a.** Flowchart of the A. baumannii search. **Figure S1b.** Flowchart of the Enterobacteriaceae search. **Figure S1c.** Flowchart of the *P. aeruginosa* search. **Figure S2.** Patient distribution by country and bacterial phenotype. **Table S3.** Summary of studies on paediatric population. **Table S4.** Microbiological information on carbapenem resistance. **Table S5.** Distribution of the resistance mechanisms in the included studies by bacterial phenotype. **Figure S3.** Quantitative data on concomitant prevalence of resistance to other antibiotic agents in CR-GNB reported by the individual studies. ANNEX III: Quantitative synthesis. ANNEX IV: Quality appraisal. **Table S6.** Quality appraisal of the non-randomized studies by domain (alphabetical order). **Table S7.** Quality appraisal of the randomized studies by domain. ANNEX V: References of the included studies.

## Data Availability

All data generated or analysed during this study are included in the manuscript and in its supplementary material.

## Abbreviations

CR-GNB: Carbapenem-resistant Gram-negative bacteria
RCT: Randomized control trial
XDR: Extensively drug-resistant
PDR: Pan drug-resistant
GARDP: Global Antibiotic Research & Development Partnership
LOS: Length of hospital stay
RoB: Risk of Bias
ICU: Intensive care unit
ROBINS-I: Risk Of Bias tool for Non-randomized Studies of Interventions
IQR: Interquartile Range
NMA: Network Meta-Analysis
PRISMA: Preferred Reporting Items Systematic Review and Meta-Analysis Protocols
HAP/VAP: Hospital- and ventilator acquired pneumonia
