# Supplementary material for: The role of combination therapy in the treatment of severe infections caused by carbapenem resistant gram-negatives: a systematic review of clinical studies
Source: BMC Infect Dis. 2021 Jun 9;21:545. doi: 10.1186/s12879-021-06253-x (PMC8188907; doi:10.1186/s12879-021-06253-x)
Supplement: Supplementary file 1 — Additional file 1: ANNEX I: Methods. Table S1. Search strings adopted for the systematic review. Table S2: Description of the employed tools for assessment of the study quality. ANNEX II: Qualitative synthesis. Figure S1a. Flowchart of the A. baumannii search. Figure S1b. Flowchart of the Enterobacteriaceae search. Figure S1c. Flowchart of the P. aeruginosa search. Figure S2. Patient distribution by country and bacterial phenotype. Table S3. Summary of studies on paediatric population. Table S4. Microbiological information on carbapenem resistance. Table S5. Distribution of the resistance mechanisms in the included studies by bacterial phenotype. Figure S3. Quantitative data on concomitant prevalence of resistance to other antibiotic agents in CR-GNB reported by the individual studies. ANNEX III: Quantitative synthesis. ANNEX IV: Quality appraisal. Table S6. Quality appraisal of the non-randomized studies by domain (alphabetical order). Table S7. Quality appraisal of the randomized studies by domain. ANNEX V: References of the included studies. [file 12879_2021_6253_MOESM1_ESM.docx]

**Supplementary Material**

**The role of combination therapy in the treatment of severe infections caused by Carbapenem Resistant Gram-Negatives. A systematic review of clinical studies**

Alessia SAVOLDI, Elena CARRARA, Laura JV PIDDOCK, Francois FRANCESCHI, Sally ELLIS, Margherita CHIAMENTI, Damiano BRAGANTINI, Elda RIGHI, Evelina TACCONELLI

**Annex I:** Methods…………………………………………………………………………………………………..…….2

**Annex II:** Qualitative synthesis…………………………………………………………………………………………..4

**Annex III:** Quantitative synthesis……………………………………………………………………………………….12

**Annex IV:** Quality appraisal……………………………………………………………………………………………..22

**Annex V:** References of the included studies……………………………………………………………………………25

**ANNEX I: Methods**

**Supplementary Table 1: Search strings adopted for the systematic review**

| **PubMed** | ((acinetobacter baumannii) AND carbapenem resistan* OR multi drug resistan* OR pan resistan*) AND (treatment OR therapy OR antibiotic OR antibacterial) |
| --- | --- |
|  | (((enterobacteriaceae OR klebsiella)) AND (carbapenem resistan* OR multi drug resistan* OR pan resistan*)) AND (treatment OR therapy OR antibiotic OR antibacterial) °° |
|  | ((pseudomonas) AND (carbapenem resistan* OR multi drug resistan* OR pan resistan*)) AND (treatment OR therapy OR antibiotic OR antibacterial) |
| **Cochrane Library** | Acinetobacter AND treatment AND carbapenem resistant |
|  | (Klebsiella OR Enterobacteriaceae) AND treatment AND carbapenem resistant |
|  | Pseudomonas AND treatment AND carbapenem resistant |
| **Clincaltrial.gov** | Acinetobacter (only completed trials) |
|  | Enterobacteriaceae/Klebsiella (only completed trials) |
|  | Pseudomonas (only completed trials) |

°°With regard to the search string related to Enterobacteriaceae, the search was started and concluded before the term “Enterobacteriaceae” came into use.

**Supplementary table 2: Description of the employed tools for assessment of the study quality**

| \| **ROBINS-I tool: Non-randomized studies** \| \| --- \| \| **CONFOUNDING** \| \| Is there any adjustment to relevant risk factors before receiving the antibiotic regimen? (i.e. baseline comorbidities, infection severity). Are methods to control for measured confounders appropriate? \| \| **SELECTION of participants** \| \| Is there inherent selection when assigning patients to one antibiotic therapy or another? (i.e. patients with poly-microbial infections receive a different antibiotic regimen during the follow-up and the outcome might change according to the other infection). \| \| **CLASSIFICATION** \| \| Does the study accurately describe the intervention (the administration of the antibiotic treatment) in terms of number type, length, dosage of the selected antibiotic agent(s)? \| \| **DEVIATION from intended intervention** \| \| The deviation from intended intervention occurs when an intervention does not reflect the usual clinical practice and might affect the outcome. (i.e. treatment is stopped/ changed (because of improvement/worsening of patient's clinical conditions). \| \| **MISSING DATA** \| \| This bias refers to the loss of patients for the outcome analysis or relevant confounders. Availability of outcome/risk factors in 90% of the enrolled population can be considered an acceptable cut-off. \| \| **SELECTION of the REPORTED RESULTS** \| \| Are results reported for all predefined outcomes in all the patient population or is the reporting limited to some outcomes or to some subgroup of the patient population? \| | \| **Cochrane RoB tool: Randomized studies** \| \| --- \| \| **SELECTION** \| \| Biased allocation to interventions due to inadequate generation of a randomized sequence or due to inadequate concealment of allocations before assignment. \| \| **PERFORMACE** \| \| Due to knowledge of the allocated interventions by participants and personnel during the study (blinding of participants and personnel). \| \| **DETECTION** \| \| Due to knowledge of the allocated interventions by outcome assessment (blinding of the outcome to the outcome assessors) \| \| **ATTRITION** \| \| Due to amount, nature, or handling of incomplete outcome data (departure from the intervention) \| \| **REPORTING** \| \| Due to selective outcome reporting \| |
| --- | --- | --- | --- | --- | --- | --- | --- | --- | --- | --- | --- | --- | --- | --- | --- | --- | --- | --- | --- | --- | --- | --- | --- | --- | --- |

**ANNEX II: Qualitative synthesis**

**Supplementary Figure 1a: Flowchart of the *A. baumannii* search**

Records identified through databases searching
(n = 1455)

- Pubmed: 1386
- Clinicaltrial.gov: 43
- Cochrane Library: 26

Full-text articles excluded, with reasons (n = 355)

- In vitro: 144
- No MDR of interest: 51
- No outcome of interest: 95
- Other study design: 35

Records excluded after abstract reviewing
(n = 1040)

Full-text articles assessed for eligibility
(n = 422)

Records screened
(n = 1462)

Records after duplicates removed
(n =1462)

Additional records identified through other sources
(n = 13)

**Supplementary Figure 1b: Flowchart of the Enterobacteriaceae search**

Studies included in quantitative synthesis (meta-analysis)
(n=67)

Studies included in the qualitative synthesis
(n = 67)

Records identified through database searching

- Pubmed: 3189
- Clinicaltrial.gov: 31
- Cochrane Library: 78
  (n = 3298)

Full-text articles excluded
(n = 805)

- In vitro: 191
- No MDR of interest: 81
- No outcome of interest: 210
- Other study design: 323

Records excluded after abstract reviewing
(n = 2439)

Studies included in quantitative synthesis (meta-analysis)
(n=49)

Studies included in the qualitative synthesis
(n =49)

Full-text articles assessed for eligibility
(n =854)

Records screened
(n = 3293)

Records after duplicates removed
(n = 3293)

Additional records identified through other sources
(n = 12)

**Supplementary Figure 1c: Flowchart of the *P. aeruginosa* search**

Records identified through database searching
(n = 1553)

- Pubmed: 1450
- Clinicaltrial.gov: 78
- Cochrane Library: 25

Records excluded after abstract reviewing
(n = 1257)

Studies included in quantitative synthesis (meta-analysis)
(n=18)

Studies included in qualitative synthesis
(n =18)

Full-text articles assessed for eligibility
(n =312)

Records screened
(n = 1569)

Records after duplicates removed
(n = 1569)

Additional records identified through other sources
(n = 16)

**Supplementary Figure 2: Patient distribution by country and bacterial phenotype**

Full-text articles excluded
(n = 294 )

- In vitro: 89
- No MDR of interest: 79
- No outcome of interest: 96
- Other study design: 30


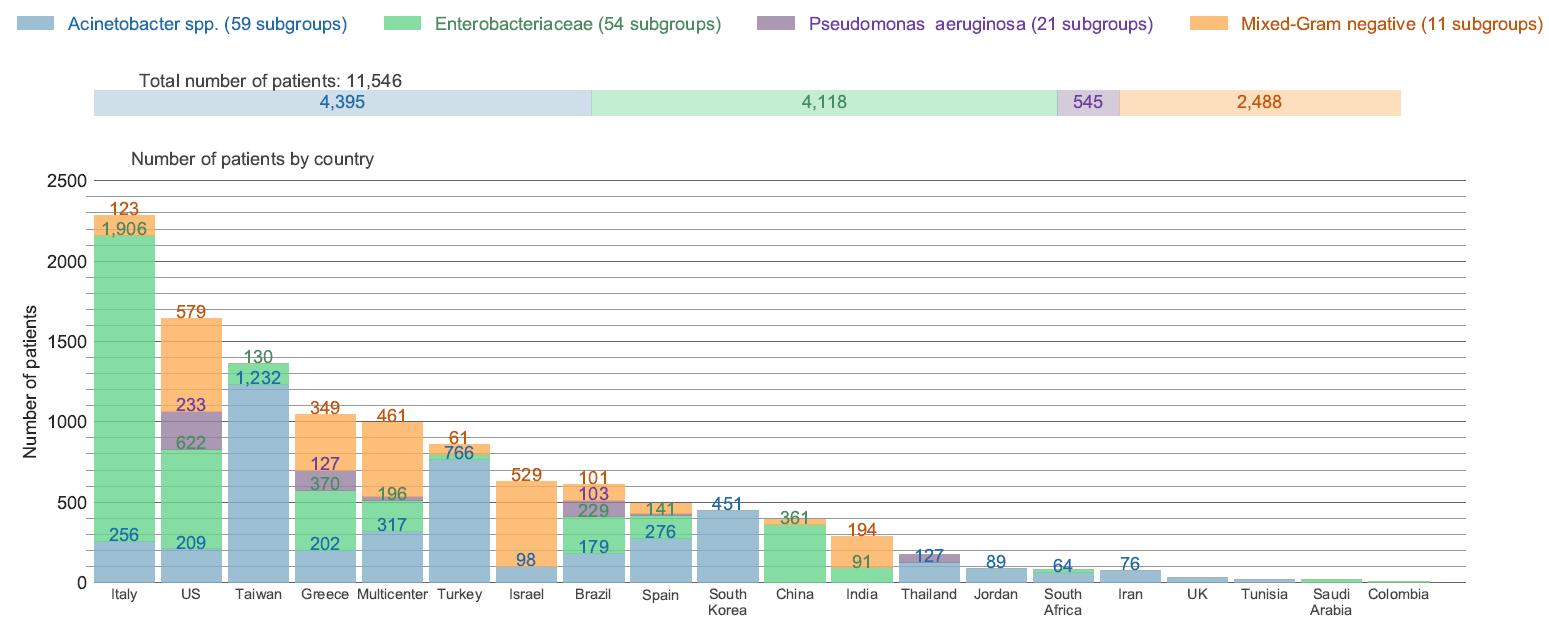


**Supplementary Table 3: Summary of studies on paediatric population.**

| **First author, publication year** | **Study design and setting** | **Infection type and bacterial phenotype** | **Antibiotic treatment** | **Comparator** | **Monomicrobial/ Polymicrobial infection** | **Outcomes and results** |
| --- | --- | --- | --- | --- | --- | --- |
| **Ceccarelli, 2015 [14]** | Case series  Monocentric (Italy)  ICU | Mixed infections  CR *A. baumannii* | Meropenem (high dosage) +  Colistin (with loading dose) +  Vancomycin | Not available | Not reported | Clinical cure 4/4  Adverse events 0/4  (Unadjusted data) |
| **Hurtado, 2012 [47]** | Retrospective case series  Monocentric (Colombia)  Hospitalwide | Mixed infections  CR  Enterobacteriaceae *(Klebsiella pneumoniae)* | Any tigecycline-containing regimen  (alone or in combination) | Not available | Yes | Clinical cure: 6/9  Adverse events: 0/9  (Unadjusted data) |
| **Karaaslan, 2016 [51]** | Retrospective  Monocentric (Turkey)  Hospitalwide | Mixed infections  CR Mixed Gram negative | Any colistin-containing regimen  (alone or in combination) | Not available | Not reported | Attributable mortality: 7/61  Adverse events: 1/61  (Unadjusted data) |

**Supplementary Table 4: Microbiological information on carbapenem resistance**

| **Breakpoint reference** | **N° of studies, (%)** | **Tool for detecting the carbapenem-resistance** | **N° of studies, (%)** |
| --- | --- | --- | --- |
| The Clinical & Laboratory Standards Institute (CLSI)* | 84 (63) | Agar dilution | 5 (4) |
|  |  | Disk diffusion | 17 (13) |
| European Committee on Antimicrobial Susceptibility Testing (EUCAST) | 23 (17) | E-test | 22 (16) |
|  |  | Broth dilution | 9 (7) |
| Not specified | 27 (20) | Automated Microdilution** | 48 (36) |
|  |  | Not specified | 33 (24) |
| **Total** | **134 (100)** | **Total** | **134 (100)** |
| *two studies referred to National Committee for Clinical Laboratory Standards (NCCLS, the former version of CLSI) | | ** Automated microdilution methods include: Vitek II, Sensititre | |

**Supplementary Table 5: Distribution of the resistance mechanisms in the included studies by bacterial phenotype**

| **Bacterial phenotype** | **Carbapenemase production** | | | **Non-carbapenemase production**  **(N° of studies)** |
| --- | --- | --- | --- | --- |
|  | **Without further information**  **(N° of studies)** | **Carbapenemase KPC type***  **(N° of studies)** | **Carbapenemase OXA type ****  **(N° of studies)** |  |
| *A. baumannii* | 1 | - | 3 | - |
| Enterobacteriaceae | 9 | 19 | 2 | 2 |
| *P. aeruginosa* | 1 | - | - | - |
| Mixed Gram Negative | 1 | 1 | - | - |
| Total | **12** | **20** | **5** | **2** |
| * mostly types 2 or 3,** mostly types 23 and 51 | | | | |

**Supplementary Figure 3: Quantitative data on concomitant prevalence of resistance to other antibiotic agents in CR-GNB reported by the individual studies**


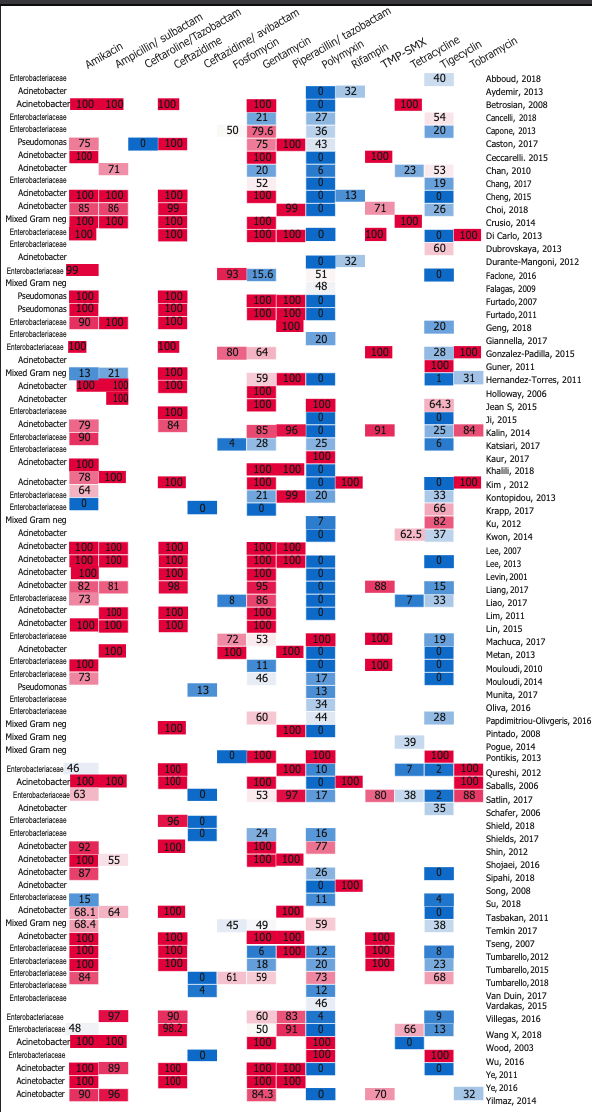


**Legend:** Decreasing intensity of red corresponds to resistance proportions from 100% (full resistance) to 50%. Decreasing intensity of blue corresponds to resistance proportions from 0% (full susceptibility) to 49%.

**ANNEX III: Quantitative synthesis**

The following procedure was applied by bacteria phenotype for the mortality, clinical cure, microbiological cure and adverse outcomes.

- ***DATA CLEANING****:* removal of “***undefined antibiotic group***”.
- ***DATA ENTRY****:* a table summarizes the number of outcome patients/total number of patients for each antibiotic regimen
- ***DATA RUN****:* network meta-analysis command. Reference treatment: Polymyxin (if not available, the reference treatment is specified in the table)
- ***DATA OUTPUT***: network geometry, that graphically represents the amount of direct and indirect evidence contributing to analysis. Irrespective of the bacterial phenotype and the outcome considered, the full closeness of the network is hampered by a variable number of disconnected components. The network geometry is reported exclusively for the outcome mortality in the result’s section of the manuscript.

***OUTCOME: MORTALITY***

**DEFINITION:** 30-day mortality. If not available, 14-day mortality or setting-related (ICU or hospital) mortality were considered.

***Bacterial phenotype: Acinetobacter baumannii***

*DATA CLEANING:* A total of 99 observations reporting data on mortality in 4078 patients with CR-*A. baumannii* infections were detected. Among them, 25 observations (1405 patients, 34%) belonged to the ***“unspecified antibiotic group”*** and therefore were removed from the analysis.

*DATA ENTRY:* 17 DIFFERENT ANTIBIOTIC REGIMENS involving 2673 patients were entered in the NMA.

*DATA OUTPUT*: three components disconnected, unable to run the NMA**.**

| **Antibiotic regimen** | **N° of observations** | **N° deaths** | **Total N° of patients** | **Mortality rate %**  **(unadjusted)** |
| --- | --- | --- | --- | --- |
| ***Bloodstream Infections*** | | | | |
| Polymyxin | 3 | 36 | 125 | 29 |
| Sulbactam | 1 | 9 | 22 | 41 |
| Carbapenem plus Polymyxin | 2 | 49 | 149 | 33 |
| Carbapenem plus Rifampin | 1 | 3 | 10 | 30 |
| Carbapenem plus Sulbactam | 2 | 36 | 60 | 60 |
| Carbapenem plus Tigecycline | 1 | 4 | 28 | 14 |
| Polymyxin plus Sulbactam | 1 | 37 | 69 | 54 |
| Polymyxin plus Tigecycline | 2 | 24 | 71 | 34 |
| ***Central nervous system infections*** | | | | |
| Polymyxin | 1 | 14 | 33 | 42 |
| Tigecycline | 1 | 4 | 6 | 67 |
| Aminoglycoside plus Tigecycline | 1 | 5 | 7 | 71 |
| Polymyxin plus Tigecycline | 1 | 5 | 10 | 50 |
| ***Ventilator-acquired pneumonia*** | | | | |
| Polymyxin | 10 | 165 | 349 | 47 |
| Sulbactam | 3 | 14 | 67 | 21 |
| Tigecycline | 2 | 49 | 107 | 46 |
| Tetracycline | 1 | 2 | 7 | 29 |
| Carbapenem plus Polymyxin | 3 | 17 | 57 | 30 |
| Polymyxin plus Rifampin | 2 | 20 | 35 | 57 |
| Polymyxin plus Tigecycline | 1 | 18 | 43 | 42 |
| Polymyxin plus Sulbactam | 4 | 60 | 98 | 61 |
| ***Mixed infections*** | | | | |
| Aminoglycosides | 1 | 6 | 12 | 50 |
| Sulbactam | 2 | 58 | 91 | 64 |
| Polymyxin | 9 | 257 | 509 | 50 |
| Tetracycline | 1 | 3 | 4 | 75 |
| Tigecycline | 4 | 61 | 100 | 61 |
| Aminoglycosides plus Sulbactam | 1 | 2 | 8 | 25 |
| Carbapenem plus Polymyxin | 2 | 88 | 182 | 48 |
| Carbapenem plus Polymyxin plus Tigecycline | 1 | 3 | 15 | 20 |
| Carbapenem plus Rifampin | 1 | 3 | 10 | 30 |
| Carbapenem plus Sulbactam | 1 | 46 | 120 | 38 |
| Polymyxin plus Glycopeptide | 1 | 14 | 29 | 48 |
| Polymyxin plus Rifampin | 5 | 86 | 212 | 41 |
| Polymyxin plus Rifampin plus Tigecycline | 1 | 5 | 19 | 26 |
| Polymyxin plus Sulbactam | 1 | 4 | 9 | 44 |

**Bacterial phenotype: Enterobacteriaceae**

*DATA CLEANING:* A total of 127 observations reporting data on mortality in 4158 patients with CR-Enterobacteriaceae infections were detected. Among them, 44 observations (2895 patients, 69%) belonged to the ***“unspecified antibiotic group”*** and therefore removed from the analysis.

*DATA ENTRY:* 26 DIFFERENT ANTIBIOTIC REGIMENS involving 1263 patients were entered in the NMA.

*DATA OUTPUT:* four components disconnected, unable to run the NMA.

| **Antibiotic regimen** | **N° of observations** | **N° deaths** | **Total N° of patients** | **Mortality rate %**  **(unadjusted)** |
| --- | --- | --- | --- | --- |
| ***Bloodstream infections*** | | | | |
| Aminoglycosides | 4 | 9 | 38 | 24 |
| Carbapenem | 4 | 16 | 55 | 29 |
| Ceftazidime/Avibactam | 1 | 1 | 13 | 8 |
| Fosfomycin | 1 | 2 | 8 | 25 |
| Polymyxin | 5 | 54 | 107 | 50 |
| Tigecycline | 4 | 33 | 74 | 45 |
| Aminoglycosides plus Carbapenem | 2 | 15 | 37 | 41 |
| Aminoglycosides plus Fosfomycin | 1 | 3 | 11 | 27 |
| Aminoglycosides plus Polymyxin | 2 | 10 | 20 | 50 |
| Aminoglycosides plus Tigecycline | 2 | 9 | 25 | 36 |
| Carbapenem plus Ertapenem | 1 | 5 | 16 | 31 |
| Carbapenem plus Polymyxin | 4 | 49 | 95 | 52 |
| Fosfomycin plus Tigecycline | 1 | 6 | 16 | 38 |
| Polymyxin plus Tigecycline | 3 | 29 | 61 | 48 |
| Carbapenem plus Polymyxin plus Tigecycline | 3 | 12 | 35 | 34 |
| Carbapenem plus Tigecycline | 2 | 11 | 20 | 55 |
| Aminoglycoside plus Carbapenem plus Tigecycline | 1 | 8 | 9 | 89 |
| Aminoglycosides plus Fosfomycin plus Tigecycline | 1 | 6 | 32 | 19 |
| ***Mixed infections*** | | | | |
| Aminoglycosides | 4 | 4 | 47 | 9 |
| Carbapenem | 2 | 5 | 18 | 28 |
| Ceftazidime/Avibactam | 1 | 13 | 39 | 33 |
| Meropenem/Vaborbactam | 1 | 5 | 32 | 16 |
| Polymyxin | 6 | 34 | 99 | 34 |
| Tigecycline | 7 | 30 | 82 | 37 |
| Trimethoprim-Sulfamethoxazole | 1 | 1 | 12 | 8 |
| Aminoglycosides plus Polymyxin | 2 | 4 | 22 | 18 |
| Aminoglycosides plus Tigecycline | 3 | 13 | 35 | 37 |
| Beta Lactam-Beta Lactam Inhibitors plus Cefepime | 1 | 5 | 24 | 21 |
| Carbapenem plus Ertapenem | 2 | 5 | 39 | 13 |
| Carbapenem plus Fosfomycin | 1 | 1 | 16 | 6 |
| Carbapenem plus Polymyxin | 1 | 8 | 39 | 21 |
| Polymyxin plus Fosfomycin | 1 | 0 | 6 | 0 |
| Polymyxin plus Tigecycline | 5 | 23 | 53 | 43 |
| Aminoglycosides plus Polymyxin plus Tigecycline | 2 | 1 | 6 | 17 |
| Carbapenem plus Ertapenem plus Polymyxin | 1 | 3 | 14 | 21 |
| Carbapenem plus Fosfomycin plus Tigecycline | 1 | 1 | 8 | 13 |

***Bacterial phenotype: Pseudomonas aeruginosa***

*DATA CLEANING:* A total of 15 observations reporting data on mortality in 410 patients with CR-*P.aeruginosa* infections were detected. Among them, 3 observations (91 patients, 22%) belonged to the ***“unspecified antibiotic group”*** and therefore removed from the analysis.

*DATA ENTRY****:*** 8 DIFFERENT ANTIBIOTIC REGIMENS involving 319 patients were entered in the NMA.

*DATA OUTPUT****:*** three components disconnected, unable to run the NMA.

| **Antibiotic regimen** | **N° of observations** | **N° deaths** | **Total N°of patients** | **Mortality rate %**  **(unadjusted)** |
| --- | --- | --- | --- | --- |
| ***Bloodstream infections*** | | | | |
| ***Polymyxin*** | 1 | 4 | 11 | 36 |
| ***Hospital- or ventilator acquired pneumonia*** | | | | |
| Polymyxin | 1 | 2 | 29 | 7 |
| Carbapenem plus Fosfomycin | 1 | 10 | 25 | 40 |
| Carbapenem plus Fosfomycin plus Polymyxin | 1 | 10 | 24 | 42 |
| ***Mixed infections*** | | | | |
| Aminoglycoside | 1 | 15 | 39 | 38 |
| Beta-Lactam / Beta-Lactamase Inhibitors | 1 | 8 | 46 | 17 |
| Cefepime | 1 | 14 | 88 | 16 |
| Ceftolozane/Tazobactam | 1 | 3 | 12 | 25 |
| Polymyxin | 3 | 9 | 37 | 24 |
| Carbapenem plus Polymyxin | 1 | 2 | 8 | 25 |

***OUTCOME: CLINICAL CURE***

***Bacterial phenotype: Acinetobacter baumannii***

*DATA CLEANING:* A total of 71 observations reporting data on clinical cure in 2892 patients with CR-*A. baumannii* infections were detected. Among them, 18 observations (986 patients, 34%) belonged to the ***“unspecified antibiotic group”*** and therefore were removed from the analysis.

*DATA ENTRY:* 17 DIFFERENT ANTIBIOTIC REGIMENS involving 1906 patients were entered in the NMA.

*DATA OUTPUT****:*** three components disconnected, unable to run the NMA.

| **Antibiotic regimen** | **N° of observations** | **N° of patients with clinical cure** | **Total N° of patients** | **Clinical cure rate%**  **(unadjusted)** |
| --- | --- | --- | --- | --- |
| ***Bloodstream infections*** | | | | |
| Polymyxin | 1 | 11 | 36 | 31 |
| Sulbactam | 1 | 6 | 12 | 50 |
| Carbapenem plus Polymyxin | 1 | 50 | 102 | 49 |
| Polymyxin plus Sulbactam | 1 | 32 | 69 | 46 |
| ***Central nervous system infections*** | | | | |
| Tigecycline | 1 | *5* | *6* | 83 |
| Aminoglycosides plus Tigecycline | 1 | 4 | 7 | **57** |
| Polymyxin plus Tigecycline | 1 | 6 | 10 | **60** |
| ***Hospital- or ventilator acquired pneumonia*** | | | | |
| Aminoglycosides | 1 | 4 | 5 | 80 |
| Polymyxine | 9 | 111 | 245 | 45 |
| Tetracycline | 2 | 15 | 18 | 83 |
| Tigecycline | 1 | 20 | 84 | 24 |
| Sulbactam | 3 | 42 | 67 | 63 |
| Aminoglycoside plus Tetracycline | 1 | 15 | 20 | 75 |
| Aminoglycoside plus Tetracycline plus Tigecycline | 1 | 3 | 4 | 75 |
| Aminoglycoside plus Tigecycline | 1 | 3 | 4 | 75 |
| Polymyxin plus Rifampicin | 1 | 11 | 21 | 52 |
| Carbapenem plus Rifampin | 1 | 2 | 10 | 20 |
| Carbapenem plus Polymyxin | 2 | 39 | 57 | 68 |
| Polymyxin plus Sulbactam | 4 | 55 | 98 | 56 |
| Polymyxin plus Tigecycline | 1 | 17 | 43 | 40 |
| ***Mixed infections*** | | | | |
| Polymyxin | 7 | 153 | 399 | 38 |
| Sulbactam | 1 | 25 | 85 | 29 |
| Tetracycline | 1 | 2 | 4 | 50 |
| Tigecycline | 3 | 19 | 33 | 58 |
| Beta Lactam Beta Lactam Inhibitor plus Polymyxin | 1 | 12 | 17 | 71 |
| Carbapenem plus Polymyxin | 3 | 146 | 300 | 49 |
| Carbapenem plus Rifampin | 1 | 2 | 10 | 20 |
| Carbapenem plus Glycopeptide plus Polymyxin | 1 | 3 | 4 | 75 |
| Polymyxin plus Glycopeptide | 1 | 17 | 29 | 59 |
| Polymyxin plus Rifampicin | 2 | 55 | 76 | 72 |
| Polymyxin plus Sulbactam | 1 | 6 | 9 | 67 |
| Polymyxin plus Tigecycline | 1 | 4 | 7 | 57 |
| Carbapenem plus Polymyxin plus Tigecycline | 1 | 10 | 15 | 67 |

***Bacterial phenotype:* Enterobacteriaceae**

*DATA CLEANING:* A total of 35 observations reporting data on clinical cure in 894 patients with CR-Enterobacteriaceae infections were detected. Among them, 10 observations (372 patients, 41%) belonged to the **“unspecified antibiotic group”** and therefore were removed from the analysis.

*DATA ENTRY:* 13 DIFFERENT ANTIBIOTIC REGIMENS involving 522 patients were entered in the NMA.

*DATA OUTPUT:* two components disconnected, unable to run NMA.

| **Antibiotic regimen** | **N° of observations** | **N° of patients with clinical cure** | **Total N° of patients** | **Clinical cure rate% (unadjusted)** |
| --- | --- | --- | --- | --- |
| ***Bloodstream infections*** | | | | |
| Aminoglycosides | 1 | 3 | 17 | 18 |
| Carbapenem | 1 | 1 | 19 | 5 |
| Ceftazidime/Avibactam | 2 | 14 | 16 | 88 |
| Tigecycline | 1 | 15 | 28 | 54 |
| Aminoglycosides plus Carbapenem | 1 | 12 | 25 | 48 |
| Carbapenem plus Ertapenem | 1 | 13 | 18 | 72 |
| Carbapenem plus Polymyxin | 2 | 19 | 48 | 40 |
| ***Mixed infections*** | | | | |
| Aminoglycosides | 2 | 13 | 27 | 48 |
| Ceftazidime/Avibactam | 3 | 42 | 65 | 65 |
| Meropenem /Vaborbactam | 1 | 21 | 32 | 66 |
| Polymyxin | 3 | 58 | 100 | 58 |
| Aminoglycosides plus Polymyxin | 1 | 11 | 17 | 65 |
| Tigecycline | 2 | 21 | 34 | 62 |
| BLBLIs plus CEFE | 1 | 14 | 24 | 58 |
| Carbapenem plus Polymyxin | 1 | 21 | 39 | 54 |
| Polymyxin plus Tigecycline | 1 | 4 | 9 | 44 |
| Aminoglycosides plus Polymyxin plus Tigecycline | 1 | 2 | 4 | 50 |

***Bacterial phenotype: Pseudomonas aeruginosa***

*DATA CLEANING:* A total of 11 observations reporting data on clinical cure in 227 patients with CR-*P. aeruginosa* infections were detected. Among them, 3 observations (97 patients, 42%) belonged to the ***“unspecified antibiotic group”*** and therefore were removed from the analysis.

*DATA ENTRY****:*** 5 DIFFERENT ANTIBIOTIC REGIMENS involving 130 patients were entered in the NMA.

*DATA OUTPUT:* two components disconnected, unable to run NMA.

| **Antibiotic regimen** | **N° of observations** | **N° of patients with clinical cure** | **Total N°of patients** | **Clinical cure rate % (unadjusted)** |
| --- | --- | --- | --- | --- |
| ***Hospital- or ventilator acquired pneumoniae*** |  |  |  |  |
| Carbapenem plus Fosfomycin | 1 | 15 | 25 | 60 |
| Carbapenem plus Fosfomycin plus Polymyxin | 1 | 14 | 24 | 58 |
| ***Mixed infections*** | | | | |
| Polymyxin | 3 | 21 | 35 | 60 |
| Beta Lactam Beta Lactam Inhibitor plus Polymyxin | 1 | 6 | 10 | 60 |
| Carbapenem plus Polymyxin | 2 | 28 | 36 | 78 |

***OUTCOME: MICROBIOLOGICAL CURE***

***Bacterial phenotype: Acinetobacter baumannii***

*DATA CLEANING:* A total of 68 observations reporting data on microbiological cure in 2623 patients with CR-*A. baumannii* infections were detected. Among them, 18 observations (1122 patients, 43%) belonged to the ***“unspecified antibiotic group”*** and therefore were removed from the analysis.

*DATA ENTRY:* 14 DIFFERENT ANTIBIOTIC REGIMENS involving 1501 patients were entered in the NMA.

*DATA OUTPUT:* three components disconnected, unable to run the NMA.

| **Antibiotic regimen** | **N° of observations** | **N° of patients with microbiological cure** | **Total N° of patients** | **Microbiological cure rate%**  **(unadjusted)** |
| --- | --- | --- | --- | --- |
| ***Bloodstream infections*** | | | | |
| Polymyxin | 1 | 20 | 36 | 56 |
| Carbapenem plus Sulbactam | 1 | 4 | 4 | 100 |
| Carbapenem plus Polymyxin | 1 | 95 | 102 | 93 |
| Polymyxin plus Sulbactam | 2 | 100 | 112 | 89 |
| ***Central nervous system infections*** | | | | |
| Tigecycline | 1 | 5 | 5 | 100 |
| Aminoglycosides plus Tigecycline | 1 | 3 | 4 | 75 |
| Polymyxin plus Tigecycline | 1 | 6 | 7 | 86 |
| ***Hospital- or ventilator acquired pneumoniae*** | | | | |
| Polymyxin | 9 | 151 | 266 | 57 |
| Sulbactam | 3 | 39 | 52 | 75 |
| Tetracycline | 1 | 4 | 5 | 80 |
| Tigecycline | 1 | 14 | 23 | 61 |
| Carbapenem plus Polymyxin | 2 | 39 | 57 | 68 |
| Carbapenem plus Rifampin | 1 | 6 | 10 | 60 |
| Polymyxin plus Rifampicin | 2 | 24 | 35 | 69 |
| Polymyxin plus Sulbactam | 4 | 68 | 92 | 74 |
| ***Mixed infections*** | | | | |
| Aminoglycosides | 1 | 17 | 32 | 53 |
| Polymyxin | 6 | 140 | 267 | 52 |
| Tetracycline | 1 | 2 | 3 | 67 |
| Tigecycline | 3 | 14 | 33 | 42 |
| Carbapenem plus Polymyxin | 1 | 14 | 120 | 12 |
| Polymyxin plus Glycopeptide | 1 | 13 | 24 | 54 |
| Polymyxin plus Rifampicin | 3 | 130 | 181 | 72 |
| Polymyxin plus Sulbactam | 1 | 6 | 9 | 67 |
| Polymyxin plus Tigecycline | 1 | 3 | 7 | 43 |
| Carbapenem plus Polymyxin plus Tigecycline | 1 | 5 | 15 | 33 |

***Bacterial phenotype*: Enterobacteriaceae**

*DATA CLEANING:* A total of 20 observations reporting data on microbiological cure in 573 patients with CR-Enterobacteriaceae infections were detected. Among them, 11 observations (388 patients, 68%) belonged to the **“unspecified antibiotic group”** and therefore were removed from the analysis.

*DATA ENTRY:* 5 DIFFERENT ANTIBIOTIC REGIMENS involving 185 patients were entered in the NMA.

*DATA OUTPUT:* four components disconnected, unable to run the NMA.

| **Antibiotic regimen** | **N° of observations** | **N° of patients with microbiological cure** | **Total N°of patients** | **Microbiological cure rate% (unadjusted)** |
| --- | --- | --- | --- | --- |
| ***Bloodstream infections*** | | | | |
| Carbapenem Plus Ertapenem | 1 | 15 | 16 | 94 |
| Carbapenem plus Polymyxin | 1 | 12 | 17 | 71 |
| Ceftazidime/Avibactam | 1 | 3 | 3 | 100 |
| ***Mixed infections*** | | | | |
| Carbapenem Plus Ertapenem | 1 | 11 | 14 | 79 |
| Ceftazidime/Avibactam | 2 | 18 | 39 | 46 |
| Meropenem/Vaborbactam | 1 | 21 | 32 | 66 |
| Polymyxin | 1 | 17 | 32 | 53 |

***Bacterial phenotype: Pseudomonas aeruginosa***

*DATA CLEANING:* A total of six observations reporting data on microbiological cure in 99 patients with CR-*P. aeruginosa* infections were detected. Among them, two observations (41 patients, 41%) belonged to the ***“unspecified antibiotic group”*** and therefore were removed from the analysis

*DATA ENTRY*: **3** DIFFERENT ANTIBIOTIC REGIMENS involving 58 patients were entered in the NMA.

*DATA OUTPUT****:*** two components disconnected, unable to run NMA.

| **Antibiotic regimen** | **N° of observations** | **N° of patients obtaining microbiological cure** | **Total N° of patients** | **Microbiological cure rate%**  **(unadjusted)** |
| --- | --- | --- | --- | --- |
| ***Hospital- or ventilator acquired pneumonia*** | | | | |
| Carbapenem plus Fosfomycin | 1 | 18 | 25 | 72 |
| Carbapenem plus Fosfomycin plus Polymyxin | 1 | 18 | 24 | 75 |
| ***Mixed infections*** | | | | |
| Ceftolozane/Tazobactam | 2 | 9 | 9 | 100 |
| Reference treatment: Ceftolozane/Tazobactam | | | | |

***OUTCOME: ADVERSE EFFECTS***

**DEFINITION:** any stage of renal disease/failure/damage as reported by each study.

***Bacterial phenotype: Acinetobacter baumannii***

*DATA CLEANING:* A total of 41 observations reporting data on renal adverse effects in 1722 patients with CR-*A. baumannii* infections were detected. Among them, 10 observations (543 patients, 31%) belonged to the ***“unspecified antibiotic group”*** and therefore were removed from the analysis.

*DATA ENTRY****:*** 11 DIFFERENT ANTIBIOTIC REGIMENS involving 1179 patients were entered in the NMA.

*DATA OUTPUT****:*** two components disconnected, unable to run NMA.

| **Antibiotic regimen** | **N° of observations** | **N° of patients with adverse effects** | **Total N° of patients** | **Adverse effects rate%**  **(unadjusted)** |
| --- | --- | --- | --- | --- |
| ***Bloodstream infections*** |  |  |  |  |
| Polymyxin | 1 | 15 | 36 | 42 |
| Carbapenem plus Polymyxin | 2 | 22 | 118 | 19 |
| Polymyxin plus Sulbactam | 1 | 10 | 69 | 14 |
| Polymyxin plus Tigecycline | 1 | 5 | 19 | 26 |
| ***Central nervous system infections*** | | | | |
| Polymyxin | 1 | 3 | 16 | 19 |
| ***Hospital- or ventilator acquired pneumoniae*** | | | | |
| Polymyxin | 4 | 47 | 142 | 33 |
| Sulbactam | 1 | 2 | 13 | 15 |
| Carbapenem plus Polymyxin | 2 | 7 | 57 | 12 |
| Polymyxin plus Rifampicin | 1 | 1 | 14 | 7 |
| Polymyxin plus Sulbactam | 2 | 3 | 43 | 7 |
| ***Mixed infections*** | | | | |
| Polymyxin | 5 | 107 | 317 | 34 |
| Sulbactam | 1 | 12 | 81 | 15 |
| Tetracycline | 1 | 0 | 4 | 0 |
| Tigecycline | 1 | 2 | 16 | 13 |
| Polymyxin plus Glycopeptide | 1 | 16 | 29 | 55 |
| Polymyxin plus Rifampicin | 3 | 52 | 177 | 29 |
| Polymyxin plus Sulbactam | 1 | 0 | 9 | 0 |
| Carbapenem plus Glycopeptide plus Polymyxin | 1 | 0 | 4 | 0 |
| Carbapenem plus Polymyxin plus Tigecycline | 1 | 3 | 15 | 20 |

**Bacterial phenotype: Enterobacteriaceae**

*DATA CLEANING:* A total of 19 observations reporting data on renal adverse observations in 545 patients with CR-Enterobacteriaceae infections were detected. Among them, 13 observations (395 patients, 72%) belonged to the **“unspecified antibiotic group”** and therefore were removed from the analysis.

*DATA ENTRY****:*** 6 DIFFERENT ANTIBIOTIC REGIMENS involving 150 patients were entered in the NMA.

*DATA OUTPUT****:*** three components disconnected, unable to run NMA.

| **Antibiotic regimen** | **N° of observations** | **N° of patients with adverse effects** | **Total N° of patients** | **Adverse effects rate% (unadjusted)** |
| --- | --- | --- | --- | --- |
| ***Bloodstream infections*** | | | | |
| Ceftazidime/avibactam | 1 | 2 | 13 | 15 |
| Aminoglycosides plus Carbapenem | 1 | 8 | 25 | 32 |
| Carbapenem plus Polymyxin | 1 | 13 | 30 | 43 |
| ***Mixed infections*** | | | | |
| Meropenem/Vaborbactam | 1 | 0 | 50 | 0 |
| Carbapenem plus Ertapenem | 1 | 3 | 18 | 17 |
| Carbapenem plus Ertapenem plus Polymyxin | 1 | 3 | 14 | 21 |
| Reference treatment: Ceftazidime/avibactam | | | |  |

***Bacterial phenotype: Pseudomonas aeruginosa***

*DATA CLEANING:* A total of seven observations reporting data on renal adverse observations in 209 patients with CR-*Pseudomonas aeruginosa* infections were detected. Among them, two observations (109 patients, 52%) belonged to the ***“unspecified antibiotic group”*** and therefore were removed from the analysis

*DATA ENTRY:* **4** DIFFERENT ANTIBIOTIC REGIMENS involving 100 patients were entered in the NMA.

*DATA OUTPUT****:*** three components disconnected, unable to run NMA.

| **Antibiotic regimen** | **N° of observations** | **N° of patients with adverse effects** | **Total N° of patients** | **Adverse effects rate%**  **(unadjusted)** |
| --- | --- | --- | --- | --- |
| ***Hospital- or ventilator acquired pnuemoniae*** | | | | |
| Polymyxin | 1 | 21 | 29 | 72 |
| Carbapenem plus Fosfomycin | 1 | 0 | 25 | 0 |
| Carbapenem plus Fosfomycin plus Polymyxin | 1 | 2 | 24 | 8 |
| ***Mixed infections*** | | | | |
| Ceftolozane/Tazobactam | 1 | 0 | 12 | 0 |
| Polymyxin | 1 | 0 | 10 | 0 |

**ANNEX IV: Quality appraisal**

**Supplementary Table 6: Quality appraisal of the non-randomized studies by domain (alphabetical order).**

| **Author, year** | **Domain 1** | **Domain 2** | **Domain 3** | **Domain 4** | **Domain 5** | **Domain 6** |
| --- | --- | --- | --- | --- | --- | --- |
|  | **CONFOUNDING** | **SELECTION of participants** | **CLASSIFICATION** of intervention | **DEVIATION** from intended intervention | **MISSING DATA** | **Bias in selection of reported results** |
| Abboud, 2018 | **Critical** | **Critical** | **Critical** | **Critical** | **Moderate** | **Low** |
| Amat, 2017 | **Critical** | **Moderate** | **Moderate** | **Moderate** | **Low** | **Low** |
| Apisarnthanarak, 2011 | **Critical** | **Critical** | **Critical** | **Moderate** | **Low** | **Low** |
| Batirel, 2014 | **Critical** | **Critical** | **Critical** | **Moderate** | **Low** | **Low** |
| Balkan, 2014 | **Critical** | **Critical** | **Critical** | **Critical** | **Low** | **Low** |
| Bassetti, 2008 | **Critical** | **Critical** | **Moderate** | **Critical** | **Low** | **Low** |
| Benattar, 2016 | **Moderate** | **Moderate** | **Critical** | **Moderate** | **Low** | **Low** |
| Britt, 2018 | **Critical** | **Critical** | **Critical** | **Critical** | **Low** | **Low** |
| Cancelli, 2018 | **Critical** | **Critical** | **Critical** | **Critical** | **Low** | **Low** |
| Capone, 2013 | **Critical** | **Critical** | **Critical** | **Moderate** | **Low** | **Low** |
| Caston, 2017 | **Critical** | **Critical** | **Critical** | **Critical** | **Low** | **Low** |
| Ceccarelli, 2015 | **Critical** | **Critical** | **Critical** | **Critical** | **Low** | **Low** |
| Chaari, 2015 | **Critical** | **Critical** | **Critical** | **Critical** | **Moderate** | **Moderate** |
| Chan, 2010 | **Critical** | **Critical** | **Critical** | **Critical** | **Moderate** | **Moderate** |
| Cheng, 2015 | **Critical** | **Critical** | **Critical** | **Critical** | **Low** | **Low** |
| Choi, 2018 | **Critical** | **Critical** | **Critical** | **Moderate** | **Low** | **Low** |
| Chang, 2017 | **Critical** | **Critical** | **Critical** | **Critical** | **Low** | **Low** |
| Chusri, 2017 | **Critical** | **Critical** | **Critical** | **Critical** | **Low** | **Low** |
| Cprek, 2016 | **Critical** | **Critical** | **Moderate** | **Critical** | **Low** | **Low** |
| Crusio, 2024 | **Critical** | **Critical** | **Critical** | **Critical** | **Low** | **Low** |
| De Oliveira, 2014 | **Critical** | **Critical** | **Critical** | **Critical** | **Low** | **Low** |
| De Pascale, 2017 | **Critical** | **Moderate** | **Moderate** | **Critical** | **Low** | **Low** |
| Di Carlo, 2013 | **Critical** | **Critical** | **Moderate** | **Critical** | **Low** | **Low** |
| Dubrovskaya, 2013 | **Critical** | **Critical** | **Critical** | **Critical** | **Low** | **Low** |
| Falagas, 2009 | **Moderate** | **Moderate** | **Critical** | **Moderate** | **Low** | **Low** |
| Falcone, 2016 | **Critical** | **Critical** | **Critical** | **Critical** | **Low** | **Low** |
| Furtado, 2007 | **Critical** | **Critical** | **Critical** | **Moderate** | **Moderate** | **Low** |
| Furtado, 2011 | **Critical** | **Moderate** | **Critical** | **Moderate** | **Low** | **Low** |
| Garbati, 2016 | **Critical** | **Critical** | **Critical** | **Critical** | **Moderate** | **Low** |
| Garnacho-Montero, 2003 | **Critical** | **Critical** | **Moderate** | **Moderate** | **Low** | **Low** |
| Garnacho-Montero, 2013 | **Critical** | **Critical** | **Critical** | **Critical** | **Low** | **Low** |
| Geng, 2018 | **Critical** | **Critical** | **Critical** | **Critical** | **Low** | **Low** |
| Ghafur, 2017 | **Critical** | **Critical** | **Critical** | **Critical** | **Moderate** | **Moderate** |
| Giannella, 2017 | **Critical** | **Critical** | **Critical** | **Moderate** | **Low** | **Low** |
| Gibson, 2016 | **Critical** | **Critical** | **Critical** | **Critical** | **Low** | **Low** |
| Gilbert, 2017 | **Critical** | **Critical** | **Critical** | **Critical** | **Moderate** | **Low** |
| Gonzalez-Padilla, 2015 | **Moderate** | **Critical** | **Critical** | **Moderate** | **Low** | **Low** |
| Gordon, 2008 | **Critical** | **Critical** | **Critical** | **Critical** | **Low** | **Low** |
| Gounden, 2008 | **Critical** | **Critical** | **Critical** | **Critical** | **Low** | **Moderate** |
| Guner, 2011 | **Critical** | **Critical** | **Critical** | **Critical** | **Moderate** | **Low** |
| Hernandez-Torres, 2011 | **Critical** | **Critical** | **Critical** | **Critical** | **Low** | **Low** |
| Holloway, 2006 | **Critical** | **Critical** | **Critical** | **Critical** | **Low** | **Low** |
| Hurtado, 2012 | **Critical** | **Critical** | **Critical** | **Critical** | **Moderate** | **Low** |
| Jean, 2015 | **Critical** | **Critical** | **Critical** | **Moderate** | **Low** | **Low** |
| Ji, 2015 | **Critical** | **Critical** | **Critical** | **Critical** | **Low** | **Low** |
| Kalin, 2014 | **Critical** | **Critical** | **Critical** | **Critical** | **Low** | **Moderate** |
| Karaaslan, 2016 | **Critical** | **Critical** | **Critical** | **Critical** | **Low** | **Low** |
| Katsiari, 2017 | **Critical** | **Critical** | **Critical** | **Critical** | **Low** | **Low** |
| Kaur, 2017 | **Critical** | **Critical** | **Critical** | **Critical** | **Low** | **Low** |
| Kim , 2012 | **Critical** | **Critical** | **Moderate** | **Critical** | **Low** | **Low** |
| Kim, 2016 | **Critical** | **Moderate** | **Moderate** | **Moderate** | **Low** | **Low** |
| King, 2017 | **Critical** | **Critical** | **Critical** | **Moderate** | **Low** | **Low** |
| Kontopidou, 2013 | **Critical** | **Critical** | **Moderate** | **Moderate** | **Low** | **Low** |
| Krapp, 2017 | **Critical** | **Critical** | **Critical** | **Critical** | **Low** | **Low** |
| Ku, 2012 | **Critical** | **Critical** | **Critical** | **Critical** | **Low** | **Low** |
| Kuo, 2009 | **Critical** | **Critical** | **Critical** | **Critical** | **Low** | **Low** |
| Kwon, 2014 | **Critical** | **Critical** | **Critical** | **Moderate** | **Low** | **Moderate** |
| Kwon, 2015 | **Critical** | **Critical** | **Critical** | **Critical** | **Moderate** | **Low** |
| Lee, 2007 | **Critical** | **Critical** | **Critical** | **Critical** | **Low** | **Low** |
| Lee, 2013 | **Critical** | **Critical** | **Critical** | **Moderate** | **Low** | **Low** |
| Levin, 2001 | **Critical** | **Critical** | **Critical** | **Critical** | **Low** | **Low** |
| Li, 2018 | **Critical** | **Critical** | **Critical** | **Critical** | **Low** | **Low** |
| Liang, 2017 | **Critical** | **Critical** | **Moderate** | **Moderate** | **Low** | **Low** |
| Liao, 2017 | **Critical** | **Critical** | **Critical** | **Critical** | **Low** | **Low** |
| Lim, 2011 | **Critical** | **Critical** | **Critical** | **Critical** | **Low** | **Low** |
| Lin, 2015 | **Critical** | **Critical** | **Critical** | **Moderate** | **Low** | **Low** |
| Lowman, 2015 | **Critical** | **Critical** | **Critical** | **Critical** | **Low** | **Low** |
| Luterbach, 2018 | **Critical** | **Critical** | **Critical** | **Critical** | **Low** | **Low** |
| Machuca, 2017 | **Critical** | **Critical** | **Critical** | **Critical** | **Low** | **Low** |
| Mastoraki, 2008 | **Critical** | **Critical** | **Critical** | **Critical** | **Low** | **Low** |
| Metan, 2010 | **Critical** | **Critical** | **Critical** | **Critical** | **Low** | **Low** |
| Michalopoulos, 2004 | **Critical** | **Critical** | **Critical** | **Critical** | **Low** | **Low** |
| Michalopoulos, 2009 | **Critical** | **Critical** | **Critical** | **Critical** | **Low** | **Low** |
| Moon, 2012 | **Critical** | **Critical** | **Critical** | **Critical** | **Low** | **Low** |
| Mouloudi, 2010 | **Critical** | **Critical** | **Critical** | **Critical** | **Low** | **Low** |
| Mouloudi, 2014 | **Critical** | **Critical** | **Critical** | **Critical** | **Low** | **Low** |
| Munita, 2017 | **Critical** | **Critical** | **Critical** | **Critical** | **Moderate** | **Low** |
| Nazer, 2015 | **Critical** | **Critical** | **Critical** | **Critical** | **Low** | **Low** |
| Nelson,2015 | **Critical** | **Critical** | **Critical** | **Critical** | **Low** | **Low** |
| Oliva, 2016 | **Critical** | **Critical** | **Critical** | **Critical** | **Low** | **Low** |
| Oliveira, 2008 | **Critical** | **Critical** | **Critical** | **Critical** | **Low** | **Low** |
| Papdimitriou-Olivgeris, 2016 | **Critical** | **Critical** | **Critical** | **Critical** | **Low** | **Low** |
| Parchem, 2016 | **Critical** | **Critical** | **Critical** | **Moderate** | **Moderate** | **Moderate** |
| Petrosillo, 2005 | **Critical** | **Critical** | **Critical** | **Critical** | **Low** | **Low** |
| Petrosillo, 2014 | **Critical** | **Critical** | **Critical** | **Critical** | **Low** | **Low** |
| Pintado, 2008 | **Critical** | **Critical** | **Critical** | **Moderate** | **Moderate** | **Moderate** |
| Pogue, 2014 | **Critical** | **Critical** | **Critical** | **Critical** | **Low** | **Low** |
| Pontikis, 2013 | **Critical** | **Critical** | **Critical** | **Critical** | **Low** | **Low** |
| Porwal, 2014 | **Critical** | **Critical** | **Critical** | **Moderate** | **Low** | **Low** |
| Qureshi, 2012 | **Critical** | **Critical** | **Critical** | **Critical** | **Low** | **Low** |
| Rigatto, 2015 | **Critical** | **Critical** | **Critical** | **Critical** | **Low** | **Low** |
| Saballs, 2006 | **Critical** | **Critical** | **Moderate** | **Moderate** | **Low** | **Low** |
| Satlin, 2017 | **Critical** | **Critical** | **Critical** | **Critical** | **Low** | **Low** |
| Schafer, 2006 | **Critical** | **Moderate** | **Moderate** | **Moderate** | **Low** | **Low** |
| Schields, 2017 | **Moderate** | **Moderate** | **Critical** | **Moderate** | **Low** | **Low** |
| Shields, 2018 | **Critical** | **Critical** | **Critical** | **Critical** | **Low** | **Low** |
| Shields, 2012 | **Critical** | **Critical** | **Moderate** | **Critical** | **Low** | **Low** |
| Shields, 2016 | **Critical** | **Critical** | **Critical** | **Critical** | **Low** | **Low** |
| Shields, 2016 | **Critical** | **Critical** | **Critical** | **Critical** | **Low** | **Low** |
| Shin, 2012 | **Critical** | **Critical** | **Critical** | **Critical** | **Low** | **Low** |
| Shojaei, 2016 | **Critical** | **Moderate** | **Critical** | **Critical** | **Low** | **Low** |
| Simsek, 2012 | **Critical** | **Critical** | **Critical** | **Critical** | **Low** | **Low** |
| Sipahi, 2018 | **Critical** | **Critical** | **Critical** | **Critical** | **Low** | **Low** |
| Song, 2008 | **Critical** | **Critical** | **Moderate** | **Moderate** | **Low** | **Low** |
| Su, 2018 | **Critical** | **Critical** | **Critical** | **Critical** | **Low** | **Low** |
| Tasbakan, 2011 | **Critical** | **Moderate** | **Critical** | **Critical** | **Low** | **Low** |
| Temkin 2017 | **Critical** | **Critical** | **Critical** | **Critical** | **Moderate** | **Moderate** |
| Tseng, 2007 | **Critical** | **Critical** | **Critical** | **Critical** | **Low** | **Low** |
| Tsung-Lin, 2018 | **Moderate** | **Moderate** | **Critical** | **Moderate** | **Low** | **Low** |
| Tumbarello, 2012 | **Critical** | **Moderate** | **Moderate** | **Critical** | **Low** | **Low** |
| Tumbarello, 2015 | **Critical** | **Critical** | **Critical** | **Critical** | **Low** | **Low** |
| Tumbarello, 2018 | **Critical** | **Critical** | **Critical** | **Moderate** | **Low** | **Low** |
| Tuon, 2018 | **Critical** | **Critical** | **Critical** | **Critical** | **Moderate** | **Moderate** |
| Van Duin, 2017 | **Critical** | **Critical** | **Critical** | **Low** | **Low** | **Low** |
| Vardakas, 2015 | **Critical** | **Critical** | **Critical** | **Critical** | **Low** | **Low** |
| Venugopalan, 2017 | **Critical** | **Moderate** | **Moderate** | **Critical** | **Low** | **Low** |
| Villegas, 2016 | **Moderate** | **Moderate** | **Critical** | **Moderate** | **Low** | **Low** |
| Wang, 2018 | **Critical** | **Moderate** | **Critical** | **Moderate** | **Low** | **Low** |
| Wood, 2003 | **Critical** | **Critical** | **Critical** | **Critical** | **Low** | **Low** |
| Wu, 2016 | **Critical** | **Critical** | **Critical** | **Critical** | **Low** | **Low** |
| Wu, 2016 | **Critical** | **Critical** | **Critical** | **Critical** | **Low** | **Low** |
| Ye, 2011 | **Critical** | **Critical** | **Critical** | **Critical** | **Low** | **Low** |
| Ye, 2016 | **Critical** | **Critical** | **Critical** | **Critical** | **Low** | **Low** |
| Yilmaz, 2014 | **Critical** | **Critical** | **Moderate** | **Moderate** | **Low** | **Low** |
| Zalts, 2013 | **Critical** | **Critical** | **Moderate** | **Critical** | **Low** | **Low** |

**Supplementary Table 7: Quality appraisal of the randomized studies by domain.**

| **Author, year** | **Domain 1** | **Domain 2** | **Domain 3** | **Domain 4** | **Domain 5** | **Domain 6** |
| --- | --- | --- | --- | --- | --- | --- |
|  | **SELECTION BIAS** | **PERFORMANCE BIAS** | **DETECTION** **BIAS** | **ATTRITION BIAS** | **REPORTING BIAS** | **OTHER BIAS** |
| Aydemir, 2013 | **Moderate** | **Moderate** | **Moderate** | **Moderate** | **Low** | **Low** |
| Betrosian, 2008 | **Critical** | **Moderate** | **Moderate** | **Critical** | **Low** | **Low** |
| Durante-Mangoni, 2012 | **Moderate** | **Critical** | **Critical** | **Moderate** | **Moderate** | **Moderate** |
| Khalili, 2018 | **Low** | **Moderate** | **Moderate** | **Low** | **Low** | **Low** |
| Makris, 2018 | **Moderate** | **Moderate** | **Moderate** | **Moderate** | **Moderate** | **Low** |
| Paul, 2018 | **Low** | **Low** | **Low** | **Low** | **Low** | **Low** |
| Sirijatuphat, 2014 | **Critical** | **Critical** | **Moderate** | **Moderate** | **Low** | **Low** |
| Wunderink, 2018 | **Moderate** | **Moderate** | **Critical** | **Moderate** | **Low** | **Low** |
| NCT01970371 | **Moderate** | **Moderate** | **Critical** | **Moderate** | **Low** | **Low** |

**ANNEX V: References of the included studies**

1. Abboud C.S., *et al.* Effect of polymyxin B-containing regimens on renal function for the treatment of carbapenem-resistant Enterobacteriacea mediastinitis. *Braz J Infect Dis*, 2018. 22(1): p. 51-54.
2. Amat T., *et al.* The combined use of tigecycline with high-dose colistin might not be associated with higher survival in critically ill patients with bacteraemia due to carbapenem-resistant Acinetobacter baumannii. *Clin Microbiol Infect*, 2018. 24(6): p. 630-634.
3. Apisarnthanarak A. and Mundy L.M. Carbapenem-resistant Pseudomonas aeruginosa pneumonia with intermediate minimum inhibitory concentrations to doripenem: combination therapy with high-dose, 4-h infusion of doripenem plus fosfomycin versus intravenous colistin plus fosfomycin*. Int J Antimicrob Agents*, 2012. 39(3): p. 271-2.
4. Aydemir H., *et al.* Colistin vs. the combination of colistin and rifampicin for the treatment of carbapenem-resistant Acinetobacter baumannii ventilator-associated pneumonia. *Epidemiol Infect,* 2013. 141(6): p. 1214-22.
5. Batirel A., *et al.* Comparison of colistin-carbapenem, colistin-sulbactam, and colistin plus other antibacterial agents for the treatment of extremely drug-resistant Acinetobacter baumannii bloodstream infections. *Eur J Clin Microbiol Infect* *Dis,* 2014. 33(8): p. 1311-22.
6. Balkan II., *et al.* Blood stream infections due to OXA-48-like carbapenemase-producing Enterobacteriaceae: treatment and survival. *Int J Infect Dis*, 2014. 26: p. 51-6.
7. Bassetti M., *et al.* Colistin and rifampicin in the treatment of multidrug-resistant Acinetobacter baumannii infections. *J Antimicrob Chemother*, 2008. 61(2): p. 417-20.
8. Benattar Y.D., et al., The Effectiveness and Safety of High-Dose Colistin: Prospective Cohort Study. Clin Infect Dis, 2016. 63(12): p. 1605-1612.
9. Betrosian A.P., *et al.* Efficacy and safety of high-dose ampicillin/sulbactam vs. colistin as monotherapy for the treatment of multidrug resistant Acinetobacter baumannii ventilator-associated pneumonia. *J Infect,* 2008. 56(6): p. 432-6.
10. Britt N.S., *et al.* Importance of Site of Infection and Antibiotic Selection in the Treatment of Carbapenem-Resistant Pseudomonas aeruginosa Sepsis. Antimicrob Agents Chemother, 2018. 62(4).
11. Cancelli F., *et al.* Role of Double-Carbapenem Regimen in the Treatment of Infections due to Carbapenemase Producing Carbapenem-Resistant Enterobacteriaceae: A Single-Center, Observational Study. *Biomed Res Int,* 2018. 2018: p. 2785696.
12. Capone A., *et al.* High rate of colistin resistance among patients with carbapenem-resistant Klebsiella pneumoniae infection accounts for an excess of mortality. *Clin Microbiol Infect,* 2013. 19(1): p. E23-e30.
13. Caston J.J., *et al.* Salvage Therapy with Ceftolozane-Tazobactam for Multidrug-Resistant Pseudomonas aeruginosa Infections. *Antimicrob Agents Chemother,* 2017. 61(3).
14. Ceccarelli G., *et al.* The role of vancomycin in addition with colistin and meropenem against colistin-sensitive multidrug resistant Acinetobacter baumannii causing severe infections in a Paediatric Intensive Care Unit. *BMC Infect Dis*, 2015. 15: p. 393.
15. Chaari A., et al. Colistin-tigecycline versus colistin-imipenem-cilastatin combinations for the treatment of Acinetobacter baumannii ventilator-acquired pneumonia: a prognosis study. *Intensive Care Med,* 2015. 41(11): p. 2018-9.
16. Chan J.D., *et al.* Antimicrobial treatment and clinical outcomes of carbapenem-resistant Acinetobacter baumannii ventilator-associated pneumonia. *J Intensive Care Med*, 2010. 25(6): p. 343-8.
17. Cheng A., *et al.* Excess Mortality Associated With Colistin-Tigecycline Compared With Colistin-Carbapenem Combination Therapy for Extensively Drug-Resistant Acinetobacter baumannii Bacteremia: A Multicenter Prospective Observational Study. *Crit Care Med,* 2015. 43(6): p. 1194-204.
18. Choi S.H., *et al.* Changes in the early mortality of adult patients with carbapenem-resistant Acinetobacter baumannii bacteremia during 11 years at an academic medical center. *J Infect Chemother,* 2019. 25(1): p. 6-11.
19. Chang Y.Y., *et al.* Clinical features of patients with carbapenem nonsusceptible Klebsiella pneumoniae and Escherichia coli in intensive care units: a nationwide multicenter study in Taiwan. *J Microbiol Immunol Infect*, 2015. 48(2): p. 219-25
20. Chusri, S., *et al.* Outcomes of adjunctive therapy with intrathecal or intraventricular administration of colistin for post-neurosurgical meningitis and ventriculitis due to carbapenem-resistant acinetobacter baumannii. *Int J Antimicrob Agents*, 2018. 51(4): p. 646-650.
21. Cprek J.B. *et al.* Ertapenem-Containing Double-Carbapenem Therapy for Treatment of Infections Caused by Carbapenem-Resistant Klebsiella pneumoniae. *Antimicrob Agents Chemother,* 2016. 60(1): p. 669-73.
22. Crusio R., et al. Epidemiology and outcome of infections with carbapenem-resistant Gram-negative bacteria treated with polymyxin B-based combination therapy. *Scand J Infect Dis,* 2014. 46(1): p. 1-8.
23. de Oliveira M.S., *et al.* Treatment of KPC-producing Enterobacteriaceae: suboptimal efficacy of polymyxins. *Clin Microbiol Infect,* 2015. 21(2): p. 179.e1-7.
24. De Pascale G., *et al*. Double carbapenem as a rescue strategy for the treatment of severe carbapenemase-producing Klebsiella pneumoniae infections: a two-center, matched case-control study. *Crit Care*, 2017. 21(1): p. 173.
25. Di Carlo P., *et al.* KPC - 3 Klebsiella pneumoniae ST258 clone infection in postoperative abdominal surgery patients in an intensive care setting: analysis of a case series of 30 patients. *BMC Anesthesiol,* 2013. 13(1): p. 13.
26. Dickstein, Y., *et al.* Treatment Outcomes of Colistin- and Carbapenem-resistant Acinetobacter baumannii Infections: An Exploratory Subgroup Analysis of a Randomized Clinical Trial. *Clin Infect Dis*, 2019. 69(5): p. 769-776.
27. Dubrovskaya Y., *et al*. Risk factors for treatment failure of polymyxin B monotherapy for carbapenem-resistant Klebsiella pneumoniae infections. *Antimicrob Agents Chemother,* 2013. 57(11): p. 5394-7.
28. Durante-Mangoni E., *et al.* Colistin and rifampicin compared with colistin alone for the treatment of serious infections due to extensively drug-resistant Acinetobacter baumannii: a multicenter, randomized clinical trial. Clin Infect Dis, 2013. 57(3): p. 349-58.
29. Falcone M., *et al.* Predictors of outcome in ICU patients with septic shock caused by Klebsiella pneumoniae carbapenemase-producing K. pneumoniae. *Clin Microbiol Infect*, 2016. 22(5): p. 444-50.
30. Falagas, M.E., *et al.* Colistin therapy for microbiologically documented multidrug-resistant Gram-negative bacterial infections: a retrospective cohort study of 258 patients. *Int J Antimicrob Agents,* 2010. 35(2): p. 194-9.
31. Furtado G.H., *et al*. Intravenous polymyxin B for the treatment of nosocomial pneumonia caused by multidrug-resistant Pseudomonas aeruginosa. *Int J Antimicrob Agents*, 2007. 30(4): p. 315-9.
32. Furtado G.H., *et al.* Prevalence and clinical outcomes of episodes of ventilator-associated pneumonia caused by SPM-1-producing and non-producing imipenem-resistant Pseudomonas aeruginosa. *Rev Soc Bras Med Trop,* 2011. 44(5): p. 604-6.
33. Garbati M.A., *et al.* Infections due to Carbapenem Resistant Enterobacteriaceae among Saudi Arabian Hospitalized Patients: A Matched Case-Control Study. *Biomed Res Int,* 2016. 2016: p. 3961684.
34. Garnacho-Montero J., *et al.* Treatment of multidrug-resistant Acinetobacter baumannii ventilator-associated pneumonia (VAP) with intravenous colistin: a comparison with imipenem-susceptible VAP. *Clin Infect Dis*, 2003. 36(9): p. 1111-8.
35. Garnacho-Montero J., *et al.* Clinical efficacy and safety of the combination of colistin plus vancomycin for the treatment of severe infections caused by carbapenem-resistant Acinetobacter baumannii. Chemotherapy, 2013. 59(3): p. 225-31.
36. Geng T.T., X. *et al.* High-dose tigecycline for the treatment of nosocomial carbapenem-resistant Klebsiella pneumoniae bloodstream infections: A retrospective cohort study. *Medicine (Baltimore),* 2018. 97(8): p. e9961.
37. Ghafur A*., et al.* Monotherapy versus combination therapy against carbapenem-resistant Gram-negative bacteria: A retrospective observational study. *Indian J Cancer,* 2016. 53(4): p. 592-594.
38. Giannella M., *et al*. Effect of combination therapy containing a high-dose carbapenem on mortality in patients with carbapenem-resistant Klebsiella pneumoniae bloodstream infection. *Int J Antimicrob Agents*, 2018. 51(2): p. 244-248.
39. Gibson G.A., *et al.* Influence of Colistin Dose on Global Cure in Patients with Bacteremia Due to Carbapenem-Resistant Gram-Negative Bacilli. *Antimicrob Agents Chemother*, 2016. 60(1): p. 431-6.
40. Gilbert B., *et al.* Evaluation of intraventricular colistin utilization: A case series. *J Crit Care*, 2017. 40: p. 161-163.
41. Gonzalez-Padilla M., *et al.* Gentamicin therapy for sepsis due to carbapenem-resistant and colistin-resistant Klebsiella pneumoniae. *J Antimicrob Chemother*, 2015. 70(3): p. 905-13.
42. Gordon N.C., *et al.* A review of clinical and microbiological outcomes following treatment of infections involving multidrug-resistant Acinetobacter baumannii with tigecycline. *J Antimicrob Chemother*, 2009. 63(4): p. 775-80.
43. Gounden R*., et al.* Safety and effectiveness of colistin compared with tobramycin for multi-drug resistant Acinetobacter baumannii infections. *BMC Infect Dis*, 2009. 9: p. 26.
44. Guner R., *et al.* Outcomes in patients infected with carbapenem-resistant Acinetobacter baumannii and treated with tigecycline alone or in combination therapy. *Infection,* 2011. 39(6): p. 515-8.
45. Hernández-Torres A., *et al*. Multidrug and carbapenem-resistant Acinetobacter baumannii infections: Factors associated with mortality. *Med Clin* (Barc), 2012. 138(15): p. 650-5.
46. Holloway K.P., *et al.* Polymyxin B and doxycycline use in patients with multidrug-resistant Acinetobacter baumannii infections in the intensive care unit. Ann Pharmacother, 2006. 40(11): p. 1939-45.
47. Hurtado I.C., *et al.* Experience with tigecycline compassionate use in pediatric patients infected with carbapenem resistant Klebsiella pneumoniae. *Rev Chilena Infectol*, 2012. 29(3): p. 317-21.
48. Jean S.S., *et al.* Comparison of the clinical efficacy between tigecycline plus extended-infusion imipenem and sulbactam plus imipenem against ventilator-associated pneumonia with pneumonic extensively drug-resistant Acinetobacter baumannii bacteremia, and correlation of clinical efficacy with in vitro synergy tests. *J Microbiol Immunol Infect,* 2016. 49(6): p. 924-933.
49. Ji S., *et al.* Cefepime combined with amoxicillin/clavulanic acid: a new choice for the KPC-producing K. pneumoniae infection. *Int J Infect Dis,* 2015. 38: p. 108-14.
50. Kalin G., *et al.* Comparison of colistin and colistin/sulbactam for the treatment of multidrug resistant Acinetobacter baumannii ventilator-associated pneumonia. *Infection*, 2014. 42(1): p. 37-42.
51. Karaaslan A., *et al.* Intravenous Colistin Use for Multidrug-Resistant Gram-Negative Infections in Pediatric Patients. *Balkan Med J,* 2016. 33(6): p. 627-632.
52. Katsiari M*., et al.* Carbapenem-resistant Klebsiella pneumoniae infections in a Greek intensive care unit: Molecular characterisation and treatment challenges. J Glob Antimicrob Resist, 2015. 3(2): p. 123-127.
53. Kaur A., *et al.,* Clinical outcome of dual colistin- and carbapenem-resistant Klebsiella pneumoniae bloodstream infections: A single-center retrospective study of 75 cases in India. Am J Infect Control, 2017. 45(11): p. 1289-1291.
54. Khalili H., *et al.* Meropenem/colistin versus meropenem/ampicillin-sulbactam in the treatment of carbapenem-resistant pneumonia. *J Comp Eff Res*, 2018. 7(9): p. 901-911.
55. Kim N.H., *et al*. Tigecycline in Carbapenem-Resistant Acinetobacter Baumannii Bacteraemia: Susceptibility and Clinical Outcome. *Scand J Infect Dis*, 2013 Apr;45(4):315-9
56. Kim W.Y., et al. Comparable Efficacy of Tigecycline versus Colistin Therapy for Multidrug-Resistant and Extensively Drug-Resistant Acinetobacter baumannii Pneumonia in Critically Ill Patients. *PLoS One*. 2016; 11(3): e0150642.
57. King M., *et al.* Multicenter Study of Outcomes with Ceftazidime-Avibactam in Patients with Carbapenem-Resistant Enterobacteriaceae Infections. *Antimicrob Agents Chemother,* 2017. 61(7).
58. Kontopidou F*., et al.* Infections Caused by Carbapenem-Resistant Klebsiella Pneumoniae Among Patients in Intensive Care Units in Greece: A Multi-Centre Study on Clinical Outcome and Therapeutic Options. *Clin Microbiol Infect,* 2014 Feb;20(2):O117-23.
59. Krapp F., *et al*. Treating complicated carbapenem resistant enterobacteriaceae infection with ceftazidime/avibactam: a retrospective study with molecular strain characterisation. *Int J Antimicrob Agents*, 2017 Jun;49(6):770-773.
60. Ku K., *et al.* Retrospective Evaluation of Colistin Versus Tigecycline for the Treatment of Acinetobacter Baumannii and/or Carbapenem-Resistant Enterobacteriaceae Infections. *Am J Infect Control,* 2012 Dec;40(10):983-7
61. Kuo S.C., *et al.* Clinical Experience With Tigecycline as Treatment for Serious Infections in Elderly and Critically Ill Patients. *J Microbiol Immunol Infect*, 2011 Feb;44(1):45-51
62. Kwon S.H., *et al*. Efficacy and Safety Profile Comparison of Colistin and Tigecycline on the Extensively Drug Resistant Acinetobacter Baumannii. *Biol Pharm Bull,* 2014;37(3):340-6.
63. Kwon K.H., *et al.* Colistin treatment in carbapenem-resistant Acinetobacter baumannii pneumonia patients: Incidence of nephrotoxicity and outcomes. *Int J Antimicrob Agents*, 2015. 45(6): p. 605-9.
64. Lee N.Y., *et al*. Combination carbapenem-sulbactam therapy for critically ill patients with multidrug-resistant Acinetobacter baumannii bacteremia: four case reports and an in vitro combination synergy study. *Pharmacotherapy*, 2007. 27(11): p. 1506-11.
65. Lee Y.T., *et al.* Clinical outcomes of tigecycline alone or in combination with other antimicrobial agents for the treatment of patients with healthcare-associated multidrug-resistant Acinetobacter baumannii infections. Eur J Clin *Microbiol Infect Dis*, 2013. 32(9): p. 1211-20.
66. Levin A.S., *et al.* Severe nosocomial infections with imipenem-resistant Acinetobacter baumannii treated with ampicillin/sulbactam. *Int J Antimicrob Agents*, 2003. 21(1): p. 58-62.
67. Li C., *et al.* Treatment options and clinical outcomes for carbapenem-resistant Enterobacteriaceae bloodstream infection in a Chinese university hospital*. J Infect Public Health,* 2019. 12(1): p. 26-31.
68. Liang C.A., *et al.* Antibiotic strategies and clinical outcomes in critically ill patients with pneumonia caused by carbapenem-resistant Acinetobacter baumannii*. Clin Microbiol Infect,* 2018. 24(8): p. 908.e1-908.e7.
69. Liao Y., *et al.* Retrospective analysis of fosfomycin combinational therapy for sepsis caused by carbapenem-resistant Klebsiella pneumoniae. *Exp Ther Med*, 2017. 13(3): p. 1003-1010.
70. Lim S.K., *et al.* The Outcomes of Using Colistin for Treating Multidrug Resistant Acinetobacter Species Bloodstream Infections. *J Korean Med Sci,* 2011 Mar;26(3):325-31
71. Lin H.S., Sulbactam Treatment for Pneumonia Involving Multidrug-Resistant Acinetobacter calcoaceticus-Acinetobacter Baumannii Complex. Infect Dis (Lond), 2015 Jun;47(6):370-8.
72. Lowman W., *et al.* Antimicrobial treatment and outcomes of critically ill patients with OXA-48like carbapenemase-producing Enterobacteriaceae infections. *Diagn Microbiol Infect Dis*, 2015. 81(2): p. 138-40.
73. Luterbach C.L., *et al.* The Role of Trimethoprim/Sulfamethoxazole in the Treatment of Infections Caused by Carbapenem-Resistant Enterobacteriaceae. *Open Forum Infect Dis*, 2019. 6(1): p. ofy351.
74. Machuca, I., *et al.* Mortality Associated with Bacteremia Due to Colistin-Resistant Klebsiella pneumoniae with High-Level Meropenem Resistance: Importance of Combination Therapy without Colistin and Carbapenems. *Antimicrob Agents Chemother,* 2017. 61(8).
75. Makris D., *et al.* Colistin versus Colistin Combined with Ampicillin-Sulbactam for Multiresistant Acinetobacter baumannii Ventilator-associated Pneumonia Treatment: An Open-label Prospective Study*. Indian J Crit Care Med,* 2018. 22(2): p. 67-77.
76. Mastoraki A., *et al*. Pseudomonas aeruginosa susceptible only to colistin in intensive care unit patients. *Surg Infect* (Larchmt), 2008. 9(2): p. 153-60.
77. Metan G., *et al.* Clinical experience with tigecycline in the treatment of carbapenem-resistant Acinetobacter infections. *J Chemother,* 2010. 22(2): p. 110-4.
78. Michalopoulos A.S., *et al.* Colistin treatment in patients with ICU-acquired infections caused by multiresistant Gram-negative bacteria: the renaissance of an old antibiotic. *Clin Microbiol Infect,* 2005. 11(2): p. 115-21.
79. Michalopoulos A*., et al.* Intravenous fosfomycin for the treatment of nosocomial infections caused by carbapenem-resistant Klebsiella pneumoniae in critically ill patients: a prospective evaluation. *Clin Microbiol Infect*, 2010. 16(2): p. 184-6.
80. Moon S.Y., *et al.* Clinical experience of tigecycline treatment in infections caused by extensively drug-resistant Acinetobacter spp. *Microb Drug Resist,* 2012. 18(6): p. 562-6.
81. Mouloudi E., *et al.* Bloodstream infections caused by metallo-beta-lactamase/Klebsiella pneumoniae carbapenemase-producing K. pneumoniae among intensive care unit patients in Greece: risk factors for infection and impact of type of resistance on outcomes. *Infect Control Hosp Epidemiol,* 2010. 31(12): p. 1250-6.
82. Mouloudi E., *et al.* Tigecycline for treatment of carbapenem-resistant Klebsiella pneumoniae infections after liver transplantation in the intensive care unit: a 3-year study. *Transplant Proc,* 2014. 46(9): p. 3219-21.
83. Munita J.M., *et al.* Multicenter Evaluation of Ceftolozane/Tazobactam for Serious Infections Caused by Carbapenem-Resistant Pseudomonas aeruginosa. *Clin Infect Dis,* 2017. 65(1): p. 158-161.
84. Nazer L.H., *et al.* High-dose colistin for microbiologically documented serious respiratory infections associated with carbapenem-resistant Acinetobacter baummannii in critically ill cancer patients: a retrospective cohort study*. Infect Dis (Lond),* 2015. 47(11): p. 755-60.
85. NCT 01970371: A Study of Plazomicin Compared With Colistin in Patients With Infection Due to Carbapenem-Resistant Enterobacteriaceae (CRE) (CARE). *Clinical Trial.gov*.
86. Nelson B.C., *et al.* Clinical outcomes associated with polymyxin B dose in patients with bloodstream infections due to carbapenem-resistant Gram-negative rods. *Antimicrob Agents Chemother*, 2015. 59(11): p. 7000-6.
87. Oliva A., *et al.* Double-carbapenem regimen, alone or in combination with colistin, in the treatment of infections caused by carbapenem-resistant Klebsiella pneumoniae (CR-Kp). J Infect, 2017. 74(1): p. 103-106.
88. Oliveira, M.S., *et al.* Ampicillin/sulbactam compared with polymyxins for the treatment of infections caused by carbapenem-resistant Acinetobacter spp. *J Antimicrob Chemother,* 2008. 61(6): p. 1369-75.
89. Papadimitriou-Olivgeris M*., et al.* Carbapenemase-producing Klebsiella pneumoniae bloodstream infection in critically ill patients: risk factors and predictors of mortality. *Eur J Clin Microbiol Infect Dis,* 2017. 36(7): p. 1125-1131.
90. Parchem N.L., *et al.* Colistin combination therapy improves microbiologic cure in critically ill patients with multi-drug resistant gram-negative pneumonia. *Eur J Clin Microbiol Infect Dis*, 2016. 35(9): p. 1433-9.
91. Petrosillo N., *et al*. Combined colistin and rifampicin therapy for carbapenem-resistant Acinetobacter baumannii infections: clinical outcome and adverse events. *Clin Microbiol Infect*, 2005. 11(8): p. 682-3.
92. Petrosillo N., et al. Clinical experience of colistin-glycopeptide combination in critically ill patients infected with Gram-negative bacteria. Antimicrob Agents Chemother, 2014. 58(2): p. 851-8.
93. Pintado V., *et al.* Intravenous colistin sulphomethate sodium for therapy of infections due to multidrug-resistant gram-negative bacteria. *J Infect*, 2008. 56(3): p. 185-90.
94. Pogue, J.M., *et al.* Carbapenem-resistance in gram-negative bacilli and intravenous minocycline: an antimicrobial stewardship approach at the Detroit Medical Center. *Clin Infect Dis*, 2014. 59 Suppl 6: p. S388-93.
95. Pontikis K., *et al.* Outcomes of critically ill intensive care unit patients treated with fosfomycin for infections due to pandrug-resistant and extensively drug-resistant carbapenemase-producing Gram-negative bacteria*. Int J Antimicrob Agents*, 2014. 43(1): p. 52-9.
96. Porwal R., *et al.* Carbapenem resistant Gram-negative bacteremia in an Indian intensive care unit: A review of the clinical profile and treatment outcome of 50 patients. *Indian J Crit Care Med*, 2014. 18(11): p. 750-3.
97. Qureshi Z.A., *et al*. Treatment outcome of bacteremia due to KPC-producing Klebsiella pneumoniae: superiority of combination antimicrobial regimens. *Antimicrob Agents Chemother*, 2012. 56(4): p. 2108-13.
98. Rigatto M.H., *et al.* Polymyxin B in Combination with Antimicrobials Lacking In Vitro Activity versus Polymyxin B in Monotherapy in Critically Ill Patients with Acinetobacter baumannii or Pseudomonas aeruginosa Infections. *Antimicrob Agents Chemother*, 2015. 59(10): p. 6575-80.
99. Saballs M., *et al.* Rifampicin/imipenem combination in the treatment of carbapenem-resistant Acinetobacter baumannii infections. *J Antimicrob Chemother,* 2006. 58(3): p. 697-700.
100. Satlin M.J., *et al*. Multicenter Clinical and Molecular Epidemiological Analysis of Bacteremia Due to Carbapenem-Resistant Enterobacteriaceae (CRE) in the CRE Epicenter of the United States. *Antimicrob Agents Chemother*, 2017 Mar 24;61(4):e02349-16
101. Schafer J.J., *et al.* Early experience with tigecycline for ventilator-associated pneumonia and bacteremia caused by multidrug-resistant Acinetobacter baumannii*. Pharmacotherapy,* 2007. 27(7): p. 980-7.
102. Shields R.K., *et al.* Epidemiology, Clinical Characteristics and Outcomes of Extensively Drug-Resistant Acinetobacter Baumannii Infections Among Solid Organ Transplant Recipients. *Infect Dis (Lond),* 2015 Jun;47(6):370-8
103. Shields R.K., *et al.* Ceftazidime-Avibactam Is Superior to Other Treatment Regimens against Carbapenem-Resistant Klebsiella pneumoniae Bacteremia. *Antimicrob Agents Chemother*, 2017. 61(8).
104. Shields, R.K.,*et al.* Pneumonia and Renal Replacement Therapy Are Risk Factors for Ceftazidime-Avibactam Treatment Failures and Resistance among Patients with Carbapenem-Resistant Enterobacteriaceae Infections. *Antimicrob Agents Chemother*, 2018. 62(5).
105. Shields R.K., *et al.* Clinical Outcomes, Drug Toxicity, and Emergence of Ceftazidime-Avibactam Resistance Among Patients Treated for Carbapenem-Resistant Enterobacteriaceae Infections. *Clin Infect Dis,* 2016. 63(12): p. 1615-1618.
106. Shields R.K., *et al.* Aminoglycosides for Treatment of Bacteremia Due to Carbapenem-Resistant Klebsiella pneumoniae. *Antimicrob Agents Chemother,* 2016. 60(5): p. 3187-92.
107. Shin J.A., *et al.* Clinical outcomes of tigecycline in the treatment of multidrug-resistant Acinetobacter baumannii infection. *Yonsei Med J,* 2012. 53(5): p. 974-84.
108. Shojaei L., *et al.* Clinical response and outcome of pneumonia due to multi-drug resistant Acinetobacter baumannii in critically ill patients. *Iran J Microbiol,* 2016. 8(5): p. 288-297.
109. Simsek F., *et al.* Colistin against colistin-only-susceptible Acinetobacter baumannii-related infections: Monotherapy or combination therapy? *Indian J Med Microbiol*, 2012. 30(4): p. 448-52.
110. Sipahi O.R., *et al.* Tigecycline in the treatment of multidrug-resistant Acinetobacter baumannii meningitis: Results of the Ege study. *Clin Neurol Neurosurg,* 2018. 172: p. 31-38.
111. Sirijatuphat R, *et al.* Preliminary study of colistin versus colistin plus fosfomycin for treatment of carbapenem-resistant Acinetobacter baumannii infections. *Antimicrob Agents Chemother*, 2014. 58(9): p. 5598-601.
112. Song J.Y., *et al.* Colistin and rifampicin combination in the treatment of ventilator-associated pneumonia caused by carbapenem-resistant Acinetobacter baumannii. *Int J Antimicrob Agents*, 2008. 32(3): p. 281-4.
113. Su C.F., *et al*. Treatment outcome of non-carbapenemase-producing carbapenem-resistant Klebsiella pneumoniae infections: a multicenter study in Taiwan. *Eur J Clin Microbiol Infect Dis*, 2018. 37(4): p. 651-659.
114. Tasbakan M.S., *et al.* Is tigecyclin a good choice in the treatment of multidrug-resistant Acinetobacter baumannii pneumonia? *J Chemother,* 2011. 23(6): p. 345-9.
115. Temkin E., *et al.* Ceftazidime-Avibactam as Salvage Therapy for Infections Caused by Carbapenem-Resistant Organisms. *Antimicrob Agents Chemother,* 2017. 61(2).
116. Tseng Y.C., *et al.* Prognosis of adult patients with bacteremia caused by extensively resistant Acinetobacter baumannii. *Diagn Microbiol Infect Dis*, 2007. 59(2): p. 181-90.
117. Tsung-Lin Y.T., *et al.* Appropriate Treatment for Bloodstream Infections Due to Carbapenem-Resistant Klebsiella pneumoniae and Escherichia coli: A Nationwide Multicenter Study in Taiwan. *Open Forum Infect Dis*, 2019. 6(2): p. ofy336
118. Tumbarello M., *et al.* Predictors of mortality in bloodstream infections caused by Klebsiella pneumoniae carbapenemase-producing K. pneumoniae: importance of combination therapy. *Clin Infect Dis,* 2012. 55(7): p. 943-50.
119. Tumbarello M., et al., Infections caused by KPC-producing Klebsiella pneumoniae: differences in therapy and mortality in a multicentre study--authors' response. *J Antimicrob Chemother,* 2015. 70(10): p. 2922.
120. Tumbarello M., *et al.* Efficacy of Ceftazidime-Avibactam Salvage Therapy in Patients With Infections Caused by Klebsiella pneumoniae Carbapenemase-producing K. pneumoniae. *Clin Infect Dis,* 2019. 68(3): p. 355-364.
121. Tuon F.F., *et al.* Polymyxin B and colistin-the economic burden of nephrotoxicity against multidrug resistant bacteria. *J Med Econ*, 2019. 22(2): p. 158-162.
122. van Duin D., *et al.* Colistin Versus Ceftazidime-Avibactam in the Treatment of Infections Due to Carbapenem-Resistant Enterobacteriaceae. *Clin Infect Dis*, 2018. 66(2): p. 163-171.
123. Vardakas K.Z., *et al.* Tigecycline for carbapenem-resistant Klebsiella pneumoniae infections in the intensive care unit. *Infect Dis (Lond),* 2015. 47(10): p. 751-3.
124. Venugopalan V., *et al.* Double carbapenem therapy (DCT) for bacteremia due to carbapenem-resistant Klebsiella pneumoniae (CRKP): from test tube to clinical practice. *Infect Dis (Lond),* 2017. 49(11-12): p. 867-870.
125. Villegas M.V., *et al.* Characterization and Clinical Impact of Bloodstream Infection Caused by Carbapenemase-Producing Enterobacteriaceae in Seven Latin American Countries. *PLoS One,* 2016. 11(4): p. e0154092.
126. Wang X., *et al.* Retrospective Observational Study from a Chinese Network of the Impact of Combination Therapy versus Monotherapy on Mortality from Carbapenem-Resistant Enterobacteriaceae Bacteremia. *Antimicrob Agents Chemother*, 2019. 63(1).
127. Wood G.C., *et al.* Tetracyclines for treating multidrug-resistant Acinetobacter baumannii ventilator-associated pneumonia. Intensive Care Med, 2003. 29(11): p. 2072-6.
128. Wu G., *et al.* Ceftazidime-Avibactam for Treatment of Carbapenem-Resistant Enterobacteriaceae Bacteremia. *Clin Infect Dis*, 2016. 63(8): p. 1147-8.
129. Wu, X., *et al.* Tigecycline Therapy for Nosocomial Pneumonia due to Carbapenem-Resistant Gram-Negative Bacteria in Critically Ill Patients Who Received Inappropriate Initial Antibiotic Treatment: A Retrospective Case Study. *Biomed Res Int,* 2016. 2016: p. 8395268.
130. Wunderink R.G., *et al*. Effect and Safety of Meropenem-Vaborbactam versus Best-Available Therapy in Patients with Carbapenem-Resistant Enterobacteriaceae Infections: The TANGO II Randomized Clinical Trial. *Infect Dis Ther,* 2018. 7(4): p. 439-455.
131. Ye J.J., *et al*. The clinical implication and prognostic predictors of tigecycline treatment for pneumonia involving multidrug-resistant Acinetobacter baumannii. *J Infect,* 2011. 63(5): p. 351-61.
132. Ye J.J., *et al*. Tigecycline-based versus sulbactam-based treatment for pneumonia involving multidrug-resistant Acinetobacter calcoaceticus-Acinetobacter baumannii complex. *BMC Infect Dis*, 2016. 16: p. 374.
133. Yilmaz G.R., *et al.* Colistin alone or combined with sulbactam or carbapenem against A. baumannii in ventilator-associated pneumonia. *J Infect Dev Ctries*, 2015. 9(5): p. 476-85
134. Zalts R., *et al.* Treatment of Carbapenem-Resistant Acinetobacter baumannii Ventilator-Associated Pneumonia: Retrospective Comparison Between Intravenous Colistin and Intravenous Ampicillin-Sulbactam. *Am J Ther*, 2016. 23(1): p. e78-85.
